# Polycystin 2 is increased in disease to protect against stress-induced cell death

**DOI:** 10.1038/s41598-019-57286-x

**Published:** 2020-01-15

**Authors:** Allison L. Brill, Tom T. Fischer, Jennifer M. Walters, Arnaud Marlier, Lorenzo R. Sewanan, Parker C. Wilson, Eric K. Johnson, Gilbert Moeckel, Lloyd G. Cantley, Stuart G. Campbell, Jeanne M. Nerbonne, Hee Jung Chung, Marie E. Robert, Barbara E. Ehrlich

**Affiliations:** 1 Department of Cellular and Molecular Physiology, Yale University, New Haven, CT 06510 United States of America; 2 Department of Pharmacology, Yale University, New Haven, CT 06510 United States of America; 3 Institute of Pharmacology, Heidelberg University, Heidelberg, Germany; 4 Department of Molecular and Integrative Physiology, University of Illinois at Urbana-Champaign, Urbana, IL 61801 United States of America; 5 Neuroscience Program, University of Illinois at Urbana-Champaign, Urbana, IL 61801 United States of America; 6 Department of Internal Medicine, Yale University, New Haven, CT 06510 United States of America; 7 Department of Biomedical Engineering, Yale University, New Haven, CT 06510 United States of America; 8 Department of Pathology, Yale University, New Haven, CT 06510 United States of America; 9 Department of Medicine, Cardiovascular Division, Washington University School of Medicine, St. Louis, MO 63110 United States of America; 10 Department of Developmental Biology, Washington University School of Medicine, St. Louis, MO 63110 United States of America; 11 Present Address: Department of Pathology and Immunology, Washington University in St. Louis, St. Louis, MO 63110 United States of America

**Keywords:** Cell biology, Physiology

## Abstract

Polycystin 2 (PC2 or TRPP1, formerly TRPP2) is a calcium-permeant Transient Receptor Potential (TRP) cation channel expressed primarily on the endoplasmic reticulum (ER) membrane and primary cilia of all cell and tissue types. Despite its ubiquitous expression throughout the body, studies of PC2 have focused primarily on its role in the kidney, as mutations in PC2 lead to the development of autosomal dominant polycystic kidney disease (ADPKD), a debilitating condition for which there is no cure. However, the endogenous role that PC2 plays in the regulation of general cellular homeostasis remains unclear. In this study, we measure how PC2 expression changes in different pathological states, determine that its abundance is increased under conditions of cellular stress in multiple tissues including human disease, and conclude that PC2-deficient cells have increased susceptibility to cell death induced by stress. Our results offer new insight into the normal function of PC2 as a ubiquitous stress-sensitive protein whose expression is up-regulated in response to cell stress to protect against pathological cell death in multiple diseases.

## Introduction

Polycystin-2 (PC2 or TRPP1, formerly TRPP2) is a Transient Receptor Potential (TRP) channel most well-known for its associated pathology. When mutated, PC2 causes autosomal dominant polycystic kidney disease (ADPKD), a debilitating condition leading to bilateral renal cyst formation and eventual kidney failure^1^. Located primarily on the endoplasmic reticulum (ER) and primary cilia of all cell and tissue types^2–5^, PC2 is a calcium (Ca^2+^)-permeant cation channel whose expression level directly affects Ca^2+^ release from the ER^5^. As such, PC2 is thought to play a key role in regulating Ca^2+^-regulated homeostasis and signaling pathways^6^. This is supported by findings showing that polycystin-deficient cells exhibit dysregulated Ca^2+^ mobilization and Ca^2+^-regulated signaling pathways^5,7^, including pathologically increased cAMP levels^8,9^ and changes in mitochondrial Ca^2+^ uptake^10,11^. The aberrant Ca^2+^ signaling caused by loss of polycystins is therefore often pointed to as a central cause of enhanced apoptosis^12,13^, excess fluid secretion^14,15^, and metabolic abnormalities^10,11,16–18^ seen in cystic kidney cells. Given the importance of PC2 in ADPKD development, most studies of PC2 have focused on its function in the kidney. However, the ubiquitous expression of PC2 in all cell types suggests that it is important in maintaining Ca^2+^ homeostasis in tissues beyond the kidney.

The tight regulation of intracellular Ca^2+^ is necessary for many physiological functions, including protecting cells against outside stressors. Oxidative and ER stress responses require Ca^2+^ influx from both the extracellular environment and intracellular stores as a key initial step for combating cell damage^19–22^. This elevated intracellular Ca^2+^ coordinates many physiological functions, such as changes in Ca^2+^-dependent gene transcription and activation of pro-survival pathways^23^. Because altered Ca^2+^ signaling occurs in cells with differential PC2 expression^5^, it follows that cells with changed PC2 levels may exhibit altered cellular homeostasis in response to stress. Indeed, beyond ADPKD, the polycystins have been linked to multiple extrarenal pathologies involving dysregulated Ca^2+^ and stress, including the development of liver^24^ and pancreatic^25^ cysts, cerebral aneurysms^26,27^, cardiac disease^28^, and cancer^29^. For instance, increased PC2 levels enhance colorectal cancer aggressiveness via modulation of cancer cell proliferation and migration through aberrant Ca^2+^-modulated signaling pathways^30^. Conversely, decreased PC2 corresponds to increased interstitial fibrosis and heightened sensitivity to injury in response to kidney ischemia^31^. It is established that the gene encoding for PC2, *Pkd2*, is differentially expressed during animal development to coordinate left-right axis determination in mice^32,33^ and zebrafish^34^. However, despite reports of altered PC2 expression in pathological states^31,35–37^, the conditions under which PC2 levels are regulated after development and into adulthood remain incompletely understood. Furthermore, how transcriptionally-regulated changes in PC2 levels influence the cellular response to stress remains unexplored. An understanding of how PC2 levels are regulated in somatic cells will give further insight into its involvement in maintaining normal cellular homeostasis, and may provide potential new targets for the treatment of ADPKD and other diseases affected by polycystin or Ca^2+^ dysfunction.

Given that increased PC2 levels correlate with enhanced cell viability and proliferation^30^, and that elevated Ca^2+^ promotes the activation of pro-survival pathways^38^, we hypothesized that PC2 expression is enhanced under conditions of stress to help protect against cell death. In this study, we found that levels of PC2 increase in disease states with ER and oxidative stress. PC2 up-regulation occurred under pathological conditions in multiple tissue types, including the kidney, liver, brain, and hearts of both humans and animal models. Finally, PC2 knock-down and knock-out cells showed increased susceptibility to stress-induced cell death. Collectively, our results indicate that PC2 acts in all tissues as a ubiquitous stress response protein whose expression levels correlate with cell survival in response to stress.

## Results

### PC2 levels are increased in kidneys with acute kidney injury

**Figure 1:**
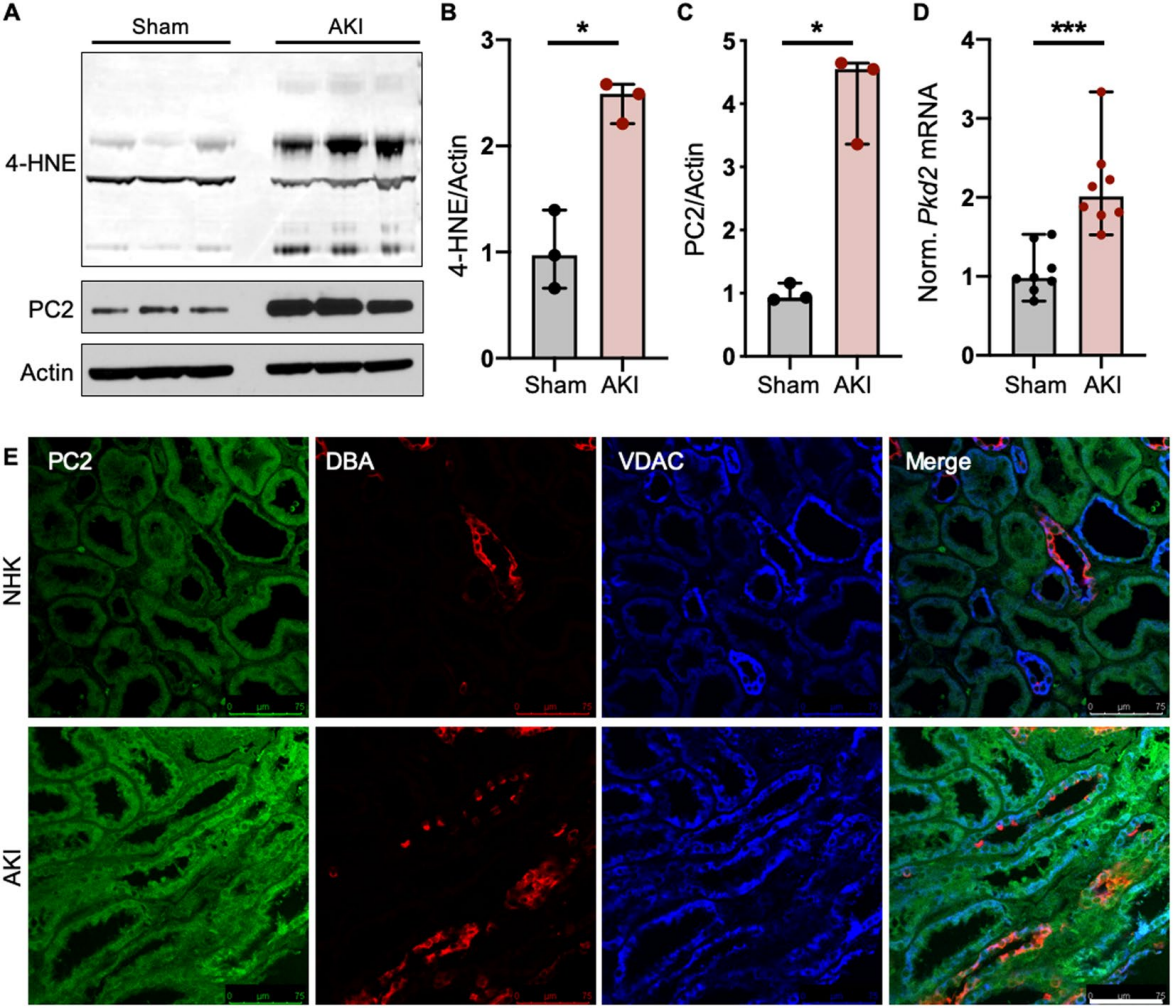
PC2 levels are increased in pathologically stressed kidneys. (**A**) Normal (Sham) and AKI-afflicted mouse kidneys were immunoblotted for 4-HNE and PC2. Each band represents one biological replicate. Full-length blots shown in Fig. S6. (**B,C**) Quantification of 4-HNE and PC2 protein abundance in Sham and AKI kidneys, normalized to actin. *p < 0.05 as determined by Mann Whitney U test. Data presented as median with range. Sample size n = 3 biological replicates per group. (**D**) Normalized mRNA expression of *Pkd2* in Sham and AKI-afflicted mouse kidneys. *Gapdh* used as internal control. Sample size n = 8 biological replicates per group. ***p < 0.001 as determined by Mann Whitney U test. Data presented as median with range. (**E**) Normal human kidneys (NHK) or kidneys with acute kidney injury (AKI) were stained for PC2 (green), DBA (red), a marker for collecting ducts, and VDAC (blue), an outer mitochondrial membrane protein. Scale bar, 75 μm.

As one of its main functions, PC2 modulates intracellular Ca^2+^ signaling. Studies demonstrate that the expression level of PC2 directly impacts the amount of ER Ca^2+^ released in response to inositol 1,4,5-trisphosphate (InsP3)-dependent stimuli^39–41^. Because kidney tubules afflicted with ischemia-induced acute kidney injury (AKI) exhibit intracellular Ca^2+^ overload and altered Ca^2+^ dynamics^42^, we tested whether the expression level of PC2 was changed in AKI-afflicted kidneys. To model injury, mice were subjected to renal unilateral ischemia/reperfusion (I/R) injury with nephrectomy in which the right renal pedicle was clamped for 27 minutes, reperfused, then sacrificed 72 hours later and the right kidney collected. Left nephrectomized kidney was used as sham control (pre-injury). Increased 4-hydroxynonenal (4-HNE) and nuclear factor kappa-light-chain-enhancer of activated B cells (NFκB), and degradation of inhibitor of kappa (IκBα) confirmed the induction of AKI in I/R kidneys compared to nephrectomy controls (Figs. 1A,B, S1A–C). Strikingly, PC2 abundance was also significantly increased in kidneys with I/R injury (Fig. 1A,C), consistent with work showing increased levels of PC2 in rat kidneys with AKI^31,35^. We then tested whether this increase was occurring transcriptionally by measuring the mRNA levels of *Pkd2* in nephrectomy control and AKI kidneys. We found that the kidneys afflicted with AKI had significantly higher levels of *Pkd2* mRNA (Fig. 1D), indicating that both PC2 transcript and protein are increased with stress. To confirm the translational relevance of this response in humans, we performed immunofluorescent staining for PC2, collecting ducts (staining for the lectin Dolichos biflorus agglutinin [DBA]), and mitochondria (staining for the voltage-dependent anion channel [VDAC]) in normal human kidneys (NHK) or kidneys from patients diagnosed with acute tubular injury (AKI; Figs. 1E, S1D; patient information included in Table S1). Quantification of PC2 intensity per cell area revealed that, as in the murine response to AKI, PC2 was significantly increased in human kidney tubules with AKI (Fig. S1E).

### PC2 is increased in livers with non-alcoholic fatty liver disease

**Figure 2:**
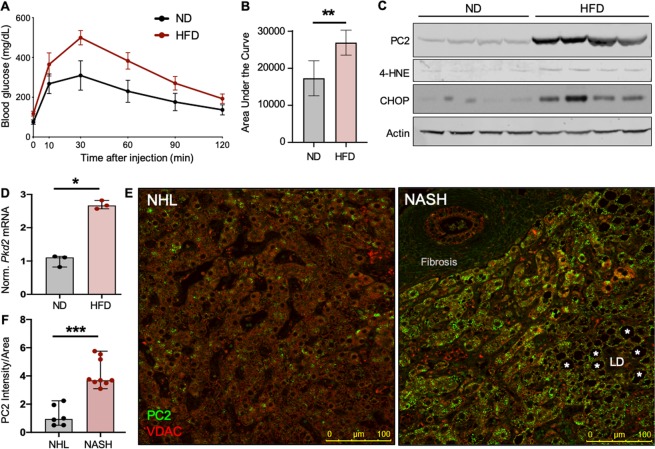
PC2 levels are increased in livers with NAFLD. (**A**) Plasma blood glucose levels and (**B**) quantified area under the curve during glucose tolerance tests of mice fed ND or HFD for 8 weeks. Shown are the mean area under the curve ± SD of 5 mice per group. **p < 0.01 as determined by Mann Whitney U test. (**C**) Mouse livers from ND- or HFD-fed mice were immunoblotted for PC2, 4-HNE, and CHOP. Each lane represents one biological replicate; sample size n = 4 per group. Full-length blots shown in Fig. S7. (**D**) Normalized mRNA expression of *Pkd2* in livers of mice fed ND or HFD for 14 months. *Gapdh* used as internal control. Sample size n = 3 biological replicates per group. *p < 0.05 as determined by Mann Whitney U test. Data presented as median with range. (**E**) Normal human livers (NHL) or livers with non-alcoholic steatohepatitis (NASH) were stained for PC2 (green) and VDAC (red). Scale bar, 100 μm. LD = lipid droplet. Asterisks depict lipid accumulation within the liver. (**F**) PC2 intensity normalized to cell area was quantified in human NHK and NASH livers. ***p < 0.001 as determined Mann Whitney U test. Quantification is of 5 images per sample; sample number NHL n = 6, NASH n = 9.

Whereas kidney cyst development with ADPKD is well-established, pathologies caused by mutations to *PKD2* do not exclusively affect the kidneys. The development of cysts arising from hepatic epithelial cells is a common extrarenal result of ADPKD^24^. Like cystic kidney cells, PC2-null cystic liver cells exhibit altered intracellular Ca^2+^ handling and changes in intracellular signaling pathways, indicating the importance of PC2 in tissues outside of the kidney^43^. To investigate whether PC2 abundance also changes in liver cells with stress, we fed mice a normal diet (ND) as control, or high-fat diet (HFD) to induce insulin resistance and hepatic stress^44^. After 8 weeks, mice were subjected to glucose tolerance tests, and the HFD-fed mice were found to be glucose intolerant compared to ND-fed mice (Fig. 2A,B). Livers from these mice were collected and showed increased levels of 4-HNE and C/EBP Homologous Protein (CHOP; Figs. 2C, S2A,B), indicating the induction of stress in HFD-fed mice. Immunoblotting for PC2 showed a significant increase in stressed livers from HFD-fed mice (Figs. 2C, S2C). Additionally, qPCR analysis of liver mRNA from mice fed ND or HFD showed a significant increase in *Pkd2* mRNA in the HFD-fed mouse livers (Fig. 2D), demonstrating that stress-related *Pkd2* up-regulation is not restricted to renal tissue.

The translational relevance of these findings was verified by performing immunofluorescent staining of normal human livers (NHL) and livers from patients with diet-induced non-alcoholic steatohepatitis (NASH; Figs. 2E, S2D; patient information included in Table S2). Quantification of PC2 fluorescence per cell area demonstrated that human livers afflicted with non-alcoholic fatty liver disease (NAFLD) likewise have increased abundance of PC2 (Fig. 2F), supporting the hypothesis that PC2 retains critical function in tissues beyond the kidney.

### PC2 is up-regulated with stress in cardiac tissue

Although the most striking symptom of ADPKD is the formation of bilateral renal cysts, the most common cause of death in ADPKD patients is a consequence of cardiac abnormalities^45,46^. Due to the expression of PC2 in cardiac tissue and the apparent importance of the polycystins in heart function, PC2 levels were examined in a murine model of cardiomyopathy and were found to be increased in isoproterenol (ISO)-treated hearts compared to healthy controls^36^. We observed that cellular stress was induced in the hearts of these ISO-treated mice as indicated by increased 4-HNE levels (Fig. S3A,B), confirming that, as in kidney and liver tissue, increased PC2 was accompanied by cellular stress in the ISO-treated hearts. To test whether PC2 was also transcriptionally up-regulated in the hearts of stressed mice, we compared mRNA levels of *Pkd2* in left ventricular (LV) tissue samples from control (sham-operated) and stressed (subjected to transverse aortic constriction [TAC]) mice^47^. Unlike the ISO-treated mice, TAC mice develop cardiac hypertrophy induced by a physical rather than chemical stressor. Compared to sham-operated mice, LV samples from TAC mice showed an increase in *Pkd2* mRNA (Fig. S3C), confirming that PC2 is up-regulated transcriptionally in stressed mouse hearts using multiple methods of stress induction.

**Figure 3:**
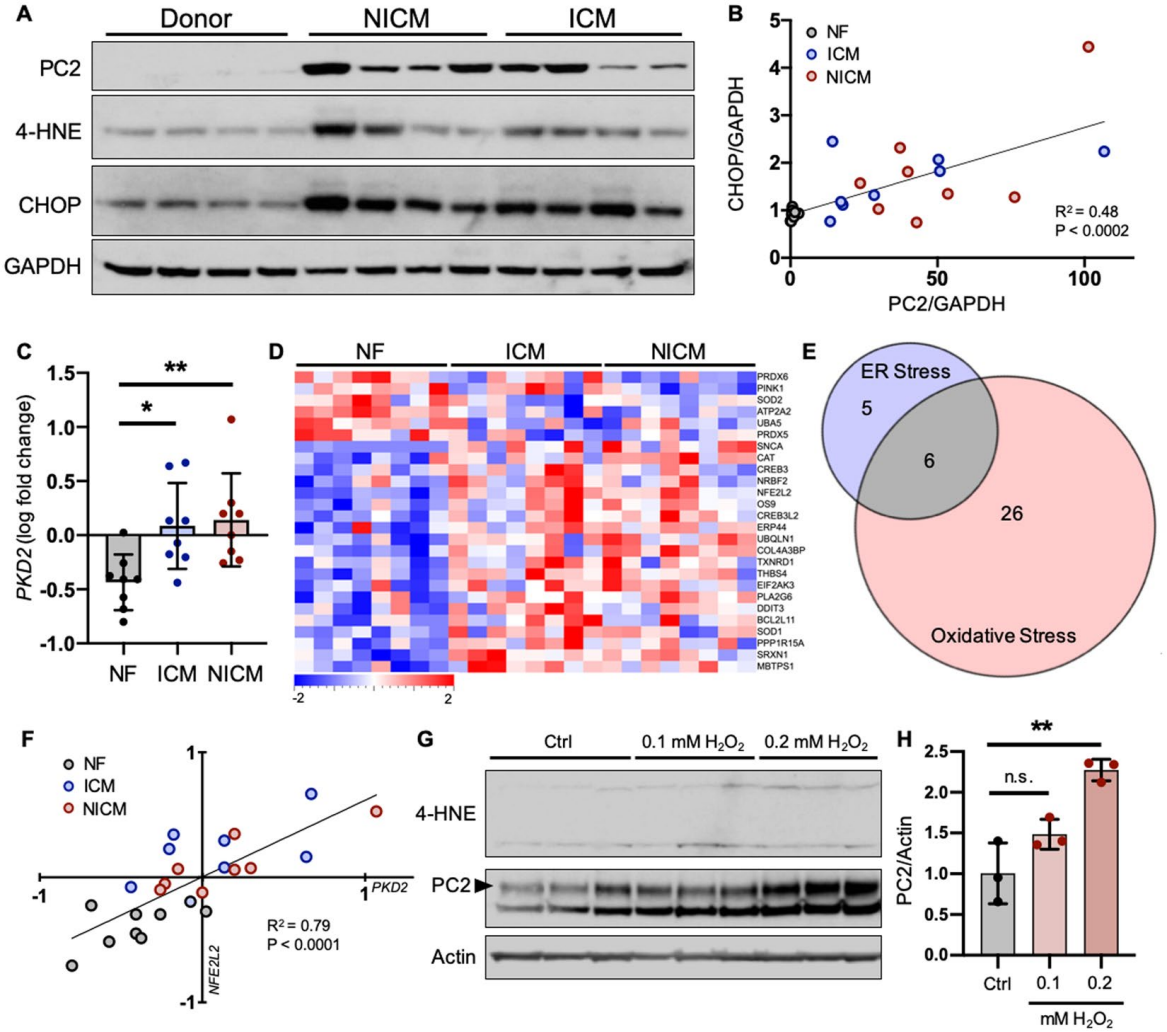
PC2 expression is up-regulated with stress and correlates with ISR pathway activation. (**A**) Left ventricular (LV) tissue samples from non-failing (NF) human hearts and from heart failure patients with non-ischemic cardiomyopathy (NICM) or ischemic cardiomyopathy (ICM) were immunoblotted for PC2, 4-HNE, and CHOP. Each lane represents one biological replicate; data shown are representative of n = 8 for each condition. Full-length blots shown in Fig. S8. (**B**) Normalized protein expression of PC2 was plotted against its corresponding normalized CHOP expression. Linear regression analysis demonstrated that PC2 levels significantly correlated with stress induction as measured by CHOP abundance. (**C**) RNA-Seq RPKM values from human NF LV samples and in heart failure patients diagnosed with ICM or NICM were log_2_ transformed and their fold change determined. *PKD2* transcripts were increased in the LV of ICM and NICM, compared to NF, hearts. *p < 0.05 and **p < 0.01 as determined by one-way ANOVA. Data presented as mean ± SD. (**D**) Curated lists of ER- and oxidative-stress associated genes were used to generate a heat map of stress-related gene expression in NF, ICM, and NICM samples. All genes shown exhibited significant differential expression (p < 0.05) amongst groups. A color legend is pictured with a scale from −2 to +2-fold change. (**E**) Genes that were overlapping between MetaCore and IPA-curated lists were used to generate ‘overlapping’ ER and ‘overlapping’ oxidative stress lists. These lists were cross-referenced, and 6 genes appeared in both lists, which were classified as ISR pathway genes. (**F**) Linear regression analysis demonstrated that one of the two ISR genes differentially expressed amongst NF, ICM, and NICM samples, *NFE2L2*, significantly correlated with *PKD2* expression. (**G**) Human iPSC-CMs were treated with increasing concentrations of H_2_O_2_ for 2 hours and immunoblotted for 4-HNE and PC2. Each lane represents one independent experiment; sample size n = 3 independent experiments per group. Full-length blots shown in Fig. S9. (**H**) Quantification of PC2 abundance in untreated (Ctrl) versus H_2_O_2_-treated iPSC-CMs. **p < 0.01 as determined by one-way ANOVA. Data presented as mean ± SD.

To determine whether up-regulation of PC2 with stress is conserved in human disease, we compared levels of PC2 in left ventricular (LV) tissue samples from non-failing (NF) human hearts and LV samples of failing human hearts, obtained from patients with ischemic cardiomyopathy (ICM) or non-ischemic cardiomyopathy (NICM; patient information included in Table S3)^48^. PC2 was largely increased in the LV samples of ICM and NICM versus NF hearts (Figs. 3A, S3D), and levels of 4-HNE and CHOP were significantly increased in NICM hearts and trending toward increased in ICM samples (Fig. S3E,F). However, because these stress markers were not significantly increased in ICM samples, we questioned whether the up-regulation of PC2 correlated to the degree of stress in these heart samples. We therefore plotted the relative protein levels of PC2 versus CHOP and found a significant correlation between the expression of these proteins (Fig. 3B), demonstrating that although statistical analyses of 4-HNE and CHOP levels do not show a significant increase in ICM samples, the degree of stress in the heart directly correlates with levels of PC2. Plotting relative PC2 versus 4-HNE levels showed a trend toward correlation, but this was not statistically significant due to an outlier in the NICM group (Fig. S3G).

To test if the increased PC2 seen in NICM and ICM LV tissues was due to differential gene expression, we compared the mRNA levels of *PKD2* in these samples versus NF LV samples. Transcript levels of *PKD2* were elevated in LV samples from ICM and NICM patients (Fig. 3C), indicating that PC2 increase with stress occurs via transcriptional up-regulation in human hearts. To see if there were differences in cellular stress levels associated with increased *PKD2* in the ICM and NICM failing hearts, curated lists of genes associated with cellular responses to ER and oxidative stress were collected from MetaCore (Clarivate Analytics) and Ingenuity Pathway Analysis (Ingenuity Systems, QIAGEN Bioinformatics). Genes from these lists were compiled (Tables S4 and 5, “Compiled”) and used to generate an expression heat map of NF, ICM, and NICM transcripts. All transcripts depicted in the heat map are significantly (p < 0.05) changed among the samples. Transcripts were sorted via hierarchical clustering and demonstrate distinct differential expression patterns in the stress genes of NF compared to ICM and NICM samples (Fig. 3D), demonstrating that up-regulation of *PKD2* correlates with altered stress response genes in human hearts.

Because elevated PC2 was associated with increases in canonical markers of both ER (CHOP) and oxidative (4-HNE) stress (Figs. 1A,B, 2C, Fig. S2A,B, Fig. 3A,B, and Fig. S3D–F), we hypothesized that *PKD2* expression was changed as part of the integrated stress response (ISR) pathway, an essential adaptive pathway that is activated in response to both ER and oxidative stressors^49–53^. To explore this idea, we cross-referenced the compiled ER and oxidative stress gene lists to develop a more stringent list of stress-associated genes (Tables S4 and 5, “Overlapping”), and cross-referenced these overlapping lists to identify genes involved in both responses. Between 11 ER stress- and 32 oxidative stress-associated genes, there were 6 genes present in both groups (Fig. 3E), two of which were differentially expressed in the NF versus ICM and NICM heart samples (Fig. S3H,I). We then used correlation analysis with a cutoff of 70% correlation or higher and discovered that one of the ISR genes, *NFE2L2*, positively correlated with *PKD2* expression (Fig. 3F; correlation = 79.2%). NFE2L2, also known as Nrf2, is a transcription factor that binds the antioxidant response element (ARE) and activates the expression of genes involved in the response to cellular insults^54–57^. The other ISR gene that was changed in ICM and NICM samples, *ATP2A2*, did not pass the 70% correlation cutoff (Fig. S3J). We tested whether these correlations were conserved in the LV from stressed mouse hearts and discovered that, like the human hearts, stressed TAC mice had decreased levels of *Atp2a2* (Fig. S3K) and a trend toward increased *Nfe2l2* (Fig. S3L). These data suggest that, unlike other stress response proteins, *PKD2* up-regulation is non-promiscuous and thus is directly associated with specific ISR pathways.

To then investigate whether PC2 expression increases as a direct consequence of cellular stress, we treated human induced pluripotent stem cell-derived cardiomyocytes (iPSC-CMs) with increasing concentrations of hydrogen peroxide (H_2_O_2_) to induce cell stress *in vitro*. H_2_O_2_ treatment led to increased levels of oxidative stress in iPSC-CMs as measured by 4-HNE (Figs. 3G, S3M). Immunoblotting for PC2 levels in iPSC-CMs showed two distinct bands, as has been previously observed^58^. It is thought that the higher band depicts the full-length, N-glycosylated form of PC2, with the lower band depicting a potentially cleaved, inactive form of PC2. Quantification of full-length PC2 levels showed an increase after 2 hours of H_2_O_2_ treatment in iPSC-CMs, establishing that increased PC2 occurs as a direct consequence of cellular insult (Fig. 3G,H).

### PC2 expression is increased in stressed brains

**Figure 4:**
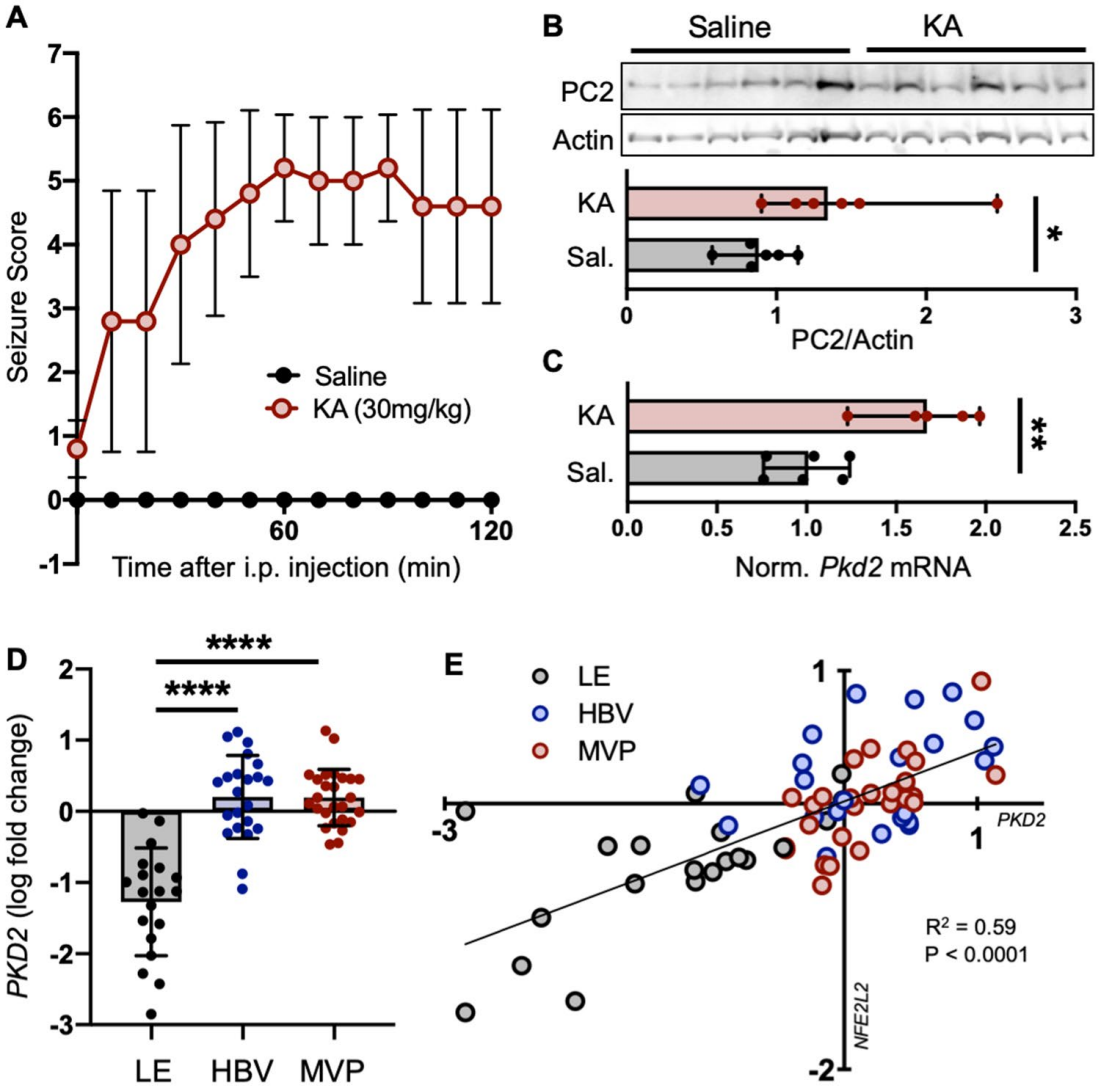
PC2 is increased in stressed brains. Mice were given injections of saline (Sal) or kainic acid (KA) to induce epileptic seizures and oxidative stress in the brain. (**A**) Seizure scores following injections were measured according to a modified Racine scale. Data shown are mean ± SD of 6 mice per group. (**B**) TOP: Hippocampal tissue was collected and immunoblotted for PC2 24 hours following injections. Each lane represents one biological replicate; sample size n = 6 per group. Full-length blots shown in Fig. S11. BOTTOM: Quantification of PC2 protein abundance in Saline and KA-treated hippocampi, normalized to actin. *p < 0.05 as determined by Mann Whitney U test. Data presented as median with range. Sample size n = 6 biological replicates per group. (**C**) Normalized mRNA expression of *Pkd2* in hippocampi from mice injected with saline or KA. *Gapdh* used as internal control. Sample number Saline n = 6, KA n = 5. **p < 0.01 as determined by Mann Whitney U test. Data presented as median with range. (**D**) RNA-Seq FPKM values from different structures (LE, leading edge; HBV, hyperplastic blood vessel; MVP, microvascular proliferation) of human glioblastoma samples were log_2_ transformed and their fold change determined. *PKD2* transcripts were increased in HBV and MVP samples compared to LE. ****p < 0.0001 as determined by one-way ANOVA. Data presented as mean ± SD. (**E**) Linear regression analysis demonstrated that the ISR gene *NFE2L2* significantly correlates with *PKD2* in human glioblastoma tissues.

Having determined that PC2 abundance changes with stress in tissues known to rely on PC2 for proper function, we questioned whether its role as a stress-sensitive protein was conserved in tissues less studied in the context of PC2. Expression of PC2 in the brain led us to test whether its abundance is also altered with stress in the central nervous system. As a model of pathologically altered Ca^2+^ signaling and cell stress in the brain^59,60^, we used kainic acid to induce acute seizures in mice^61^. Systemic injections of kainate cause epileptiform seizures in the hippocampus and induce hippocampus-restricted neuropathology comparable to that seen in patients with temporal lobe epilepsy^61^. Mice were injected intraperitoneally with saline (Sal) or kainic acid (KA; Fig. S4A), and their seizures scored according to a modified Racine scale every 10 minutes for 120 minutes (Fig. 4A, Table S6). Hippocampal lysates collected from randomly-paired, weight- and age-matched mice were found to have increased expression of PC2 in epileptic compared to saline-treated brains (Fig. 4B). *Pkd2* and *Nfe2l2* mRNA expression was likewise tested from hippocampi of saline- and KA-treated mice and found to be significantly increased with KA induction of epileptic seizures (Figs. 4C, S4B).

We then determined whether this increase was translatable to human disease by investigating if PC2 expression correlates with stress in human brains. Using the RNA-Seq dataset provided by the Allen Institute Ivy Glioblastoma Atlas Project^62^, we examined *PKD2* expression in 3 different glioblastoma structures (hyperplastic blood vessels, microvascular proliferation, and leading edge; patient information included in Table S7). Leading edge (LE) samples showed relatively low amounts of *PKD2* expression, whereas hyperplastic blood vessels (HBV) and microvascular proliferation (MVP) samples expressed relatively high amounts of *PKD2* (Fig. 4D). These findings correlated with differential expression of the ISR genes *NFE2L2* and *ATP2A2* in the different glioblastoma structures (Fig. S4C,D), indicating altered ISR levels amongst these structures. We then used correlation analysis (correlation ≥ 70%) and found that, like in human hearts, *PKD2* levels positively correlated with *NFE2L2* in human brains (Fig. 4E; correlation = 76.8%), but not with *ATP2A2* (Fig. S4E).

### PC2 up-regulation protects cells against stress-induced cell death

**Figure 5:**
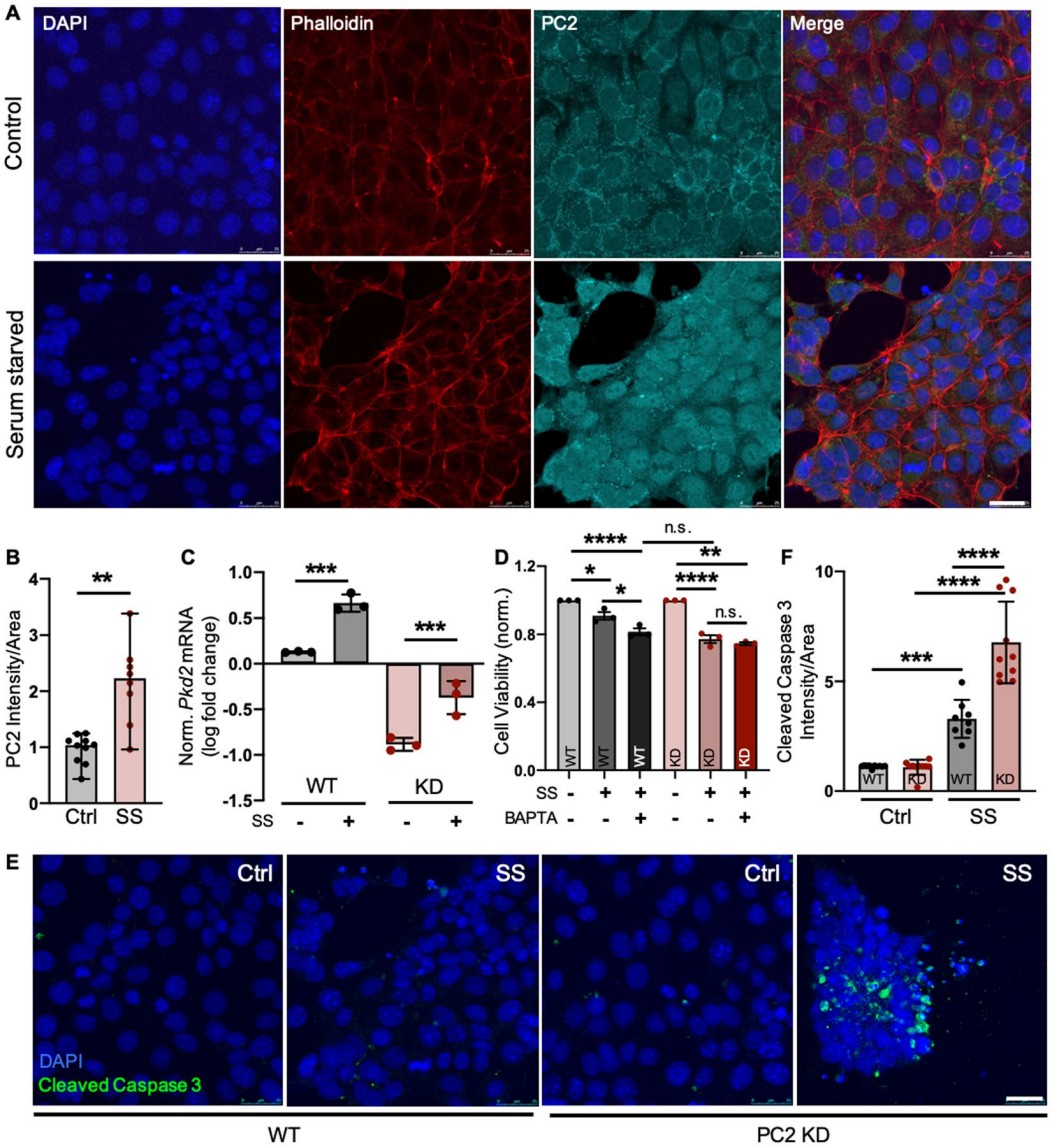
PC2 protects against stress-induced cell death. (**A**) Kidney epithelial LLC-PK1 cells were grown in normal culture medium (Control) or base medium not supplemented with FBS (Serum Starved, SS) for 24 hours, then stained for DAPI (blue), phalloidin (red), and PC2 (cyan). Scale bar, 25 μm. (**B**) PC2 intensity normalized to cell area was quantified in control and serum-starved (SS) LLC-PK1 cells. **p < 0.01 as determined by Mann Whitney U test. Quantification is of 5 images per sample; n = 10 samples per group. Data presented as median with range. (**C**) Fold change mRNA expression of *Pkd2* in serum-starved WT, and control and serum-starved PC2 KD cells compared to WT control cells. *Actin* used as internal control. n = 3 independent experiments per group. ***p < 0.001 as determined by one-way ANOVA. Data presented as mean ± SD. (**D**) Viability of WT and PC2 KD (KD) cells was tested via CellTiter-Glo assay under normal (Ctrl) and stressed (SS) conditions with or without 24-hour co-treatment of 100 nM BAPTA-AM (BAPTA). *p < 0.05, **p < 0.01, and ****p < 0.0001 as determined by one-way ANOVA. n = 3 independent experiments per group with 10 replicates per experiment. Data presented as mean ± SD. (**E**) WT and PC2 KD cells were grown in normal culture medium (Ctrl) or serum starved (SS) for 24 hours, then stained for DAPI (blue) and cleaved caspase 3 (green). Images shown are representative of 10 control and 10 serum-starved samples from both WT and PC2 KO cell lines. Scale bar, 25 μm. (**F**) Cleaved caspase 3 intensity normalized to cell area was quantified. ***p < 0.001 and ****p < 0.0001 as determined by one-way ANOVA. Quantification is of 5 images per sample; n = 10 samples per group. Data presented as mean ± SD.

Although we determined that PC2 expression is up-regulated as a consequence of cellular insult in multiple tissue types, whether this increase is detrimental or favorable for the cell remained unclear. To test this, we utilized a kidney epithelial cell line (LLC-PK1) with wild-type levels of PC2 (WT) or PC2 knocked down (PC2 KD; Fig. S5A) to assess PC2’s role in the cellular stress response. Cells were serum-starved (SS) to induce stress (Fig. S5A,B)^63–65^ and, consistent with our findings in animal and human tissues, cells showed increased PC2 mRNA and protein expression following serum starvation (Fig. 5A–C, S5A,C) and treatment with tunicamycin (TM), an inducer of ER stress (Fig. S5D–F). We then measured cell viability of WT and PC2 KD cells under basal and stressed conditions. When stress was induced through serum starvation or with TM treatment, PC2 KD cells showed decreased cell viability compared to WT cells (Figs. 5D, S5G), consistent with previous work showing that loss of PC2 renders kidney cells hyper-sensitive to stress^31^, and indicating that up-regulation of PC2 occurs as a protective mechanism. To test our hypothesis that PC2 up-regulation protects against cell stress by enhancing intracellular Ca^2+^ signaling, we measured cell viability in serum-starved cells treated concurrently with the intracellular Ca^2+^ chelator BAPTA-AM. When Ca^2+^ signaling was blunted in SS WT cells through BAPTA-AM treatment, cell viability decreased to levels of SS PC2 KD cells (Fig. 5D). However, in SS KD cells treated with BAPTA-AM, there was no further decrease in cell viability (Figs. 5D, S5H), indicating that a PC2-dependent Ca^2+^ response plays a significant role in the cells’ ability to combat stress. Finally, we measured the degree of stress-induced apoptosis through immunofluorescence and western blot analyses of cleaved caspase-3 activation and found that, compared to WT cells, PC2 KD cells had increased levels of apoptosis when stressed (Figs. 5CE,F, S5A,D,I). The same results were observed when testing another cell line (mIMCD-3) containing normal levels of PC2 (WT) or with PC2 knocked out via CRISPR/Cas9 (PC2 KO; Fig. S5J–L). These data demonstrate that PC2 up-regulation serves to protect cells against stress, and that with inadequate levels of PC2, cells are rendered hyper-susceptible to stress-induced cell death.

## Discussion

In this study, we establish that PC2 is a stress-responsive protein whose expression is elevated in a variety of cell types under multiple pathological conditions. Its up-regulation is a direct consequence of ER and oxidative stress, and its mRNA expression significantly correlates with *Nfe2l2*, a transcription factor that is part of the ISR pathway^66^. Loss of PC2 resulted in a Ca^2+^-mediated decrease in cell viability in response to stress and increased levels of stress-induced apoptosis. Collectively, these findings demonstrate that PC2 plays an important role in general cell homeostasis in numerous tissue types, and that up-regulation of PC2 helps protect cells against stress-induced cell death under pathological conditions.

The importance of PC2 in the maintenance of cellular homeostasis is well-supported by the pathological dysfunctions that occur in cystic ADPKD cells. Our findings indicate that PC2 is increased transcriptionally with cell stress, and that this occurs in response to specific signaling pathways. The function of PC2 as a modulator of Ca^2+^ signaling fits well with these findings, as Ca^2+^ mobilization is a critical step in the cell’s ability to respond to and protect against pathological stressors. As our data indicate, enhanced Ca^2+^ signaling facilitated by PC2 following cell stress contributes significantly to the cell’s adaptive response, whereas blunted Ca^2+^ through intracellular chelation or the absence of PC2 causes hypersensitivity of the cells to outside stressors. Though changes in PC2 levels have been noted in different pathological conditions, the causes for its differential expression after development remained incompletely explored. In support of our findings, previous studies have shown that both *Pkd2* transcript and protein levels are increased in kidneys following ischemia/reperfusion injury, and that mice with decreased levels of PC2 are more sensitive to injury in both kidney and smooth muscle cells^31,67–69^. The correlation we discovered between *PKD2* and *NFE2L2* levels suggests that *PKD2* may be up-regulated via Nrf2, a transcription factor that is increased following cellular insults including oxidative stress^70^. Much of what is known regarding the transcriptional regulation of *Pkd2* is based upon its cis-acting promoter region, which contains putative binding sites for developmentally-regulated transcription factors such as Sp1, NF-1, and AP-2^71,72^. However, trans-regulatory effects on *Pkd2* expression have not been described and may contain additional regulatory elements to alter PC2 levels.

Our results demonstrate that PC2 KD and KO renders cells susceptible to stress-induced death. It therefore logically follows that cystic cells characterized by loss the of PC2 would exhibit decreased cell viability in response to stress. In support of this, a standard phenotype associated with ADPKD is enhanced apoptosis during cyst development^69,73,74^, where cellular stress levels are high due to many pathological factors, including renal ischemia and altered cell metabolism. In fact, treatment of ADPKD mice with caspase inhibitors (inhibitors of apoptosis) results in decreased cyst volume and cell proliferation^74^. Additionally, PC2 haploinsufficient (*Pkd2*^*+/-*^) kidney cells that express decreased overall levels of PC2 show hypersensitivity to cell stress. *Pkd2*^+/-^ mice develop larger, more pronounced kidney cysts when treated with Tumor Necrosis Factor alpha (TNFα, an inflammatory cytokine) to induce stress as compared to wild-type littermates, whereas treatment of *Pkd2*^*+/-*^ mice with TNFα inhibitor blocked cyst formation^75^. Additional studies seeking effective treatment for kidney disease have identified resveratrol, a potent activator of Nrf2^76^, as a potentially compelling therapeutic agent for slowing cyst progression in ADPKD^77,78^. Whereas these studies have focused on the effects of resveratrol in ameliorating the inflammatory response associated with cyst development, it is possible that resveratrol may act through Nrf2 to help restore normal ISR activity in these cells as well.

Although renal cyst formation and kidney failure are arguably the most well-known symptoms of PC2 dysfunction, multiple extrarenal pathologies are also known to occur in patients with loss-of-function *PKD2* mutations. Indeed, ADPKD patients commonly present with numerous manifestations of the disease, including colonic diverticula^79,80^, hernia^81,82^, cerebral aneurisms^83^, and the development of hepatic^84^, pancreatic^25^, and seminal vesicle^85,86^ cysts. Most notably, however, rather than renal failure, the major cause of death in patients with ADPKD is due to cardiac dysfunction^83^. Studies seeking to elucidate how the loss of PC2 affects the heart have shown that with increased age, decreased PC2 levels result in cardiac dysfunction independent of renal abnormalities^87^. Additionally, in agreement with our findings, PC2 levels are enhanced in cardiomyocytes with cardiac injury, and *Pkd2*^*+/-*^ mice show a diminished capacity to recover from injury following the cessation of stress^36^. It has been suggested that the extrarenal manifestations seen in ADPKD patients are merely secondary effects caused by the main pathology of renal cysts. However, our findings highlight the importance of PC2 as a ubiquitous protein essential for proper cell function not only in the kidney, but in all tissue types. Based on this, the role of PC2 in numerous Ca^2+^-associated stress pathologies may prove to be a promising target by which to restore normal Ca^2+^ and/or ISR homeostasis.

Our *in vitro* data suggest that PC2 regulation of Ca^2+^ signaling is required to maintain cell viability in the face of stress. Whereas the function of PC2 as a Ca^2+^-permeable cation channel is well-established, there currently exists controversy over where PC2 exerts its intrinsic channel function. It is thought that due to the ER retention signal within its C-terminal tail, the majority of PC2 is located on the ER^5,88^, with the rest being localized to the primary cilia in association with PC1^89^ or TRPV4^90^, where it exerts its anti-cystogenic function^91^. However, others have suggested that PC2 is also present and functions as a Ca^2+^ channel on the plasma membrane^92,93^. In the context of our findings, we believe that PC2 exerts its stress response function from its localization on the ER based upon (1) immunofluorescent imaging demonstrating that PC2 localization remains intracellular, even under stressed conditions (Figs. S1D and S2D) and (2) the up-regulation of PC2 with stress in terminally-differentiated neurons and cardiomyocytes, whose cilia are stumpy, essentially vestigial organelles. Nevertheless, the localization of PC2 does not ultimately influence the findings of this manuscript, as PC2 is believed to facilitate enhanced cytoplasmic Ca^2+^ signaling whether functioning on the ER, primary cilia, or plasma membrane.

Additionally, though PC2 is itself can form a Ca^2+^-permeable cation channel, it is known to interact with other cation channels to enhance intracellular Ca^2+^ signaling (including the inositol 1,4,5-trisphosphate receptor^39,40,94^, ryanodine receptor^95^, and other TRP channels^90,96^). Therefore, whether PC2 is altering intracellular Ca^2+^ signaling through its intrinsic channel activity or through interactions with other channels remains unclear. Interestingly, PC2 channel activity has been shown to be sensitive to H_2_O_2_, whose treatment caused an up-regulation of PC2 in human iPSC-CMs. Because the reported IC_50_ of this inhibition is ~193 μM^97^, we do not believe that the H_2_O_2_ concentration used in our experiment (up to 200 μM) would be sufficient to completely eliminate a Ca^2+^ current through PC2. Nonetheless, if it were the case that PC2 is fully inhibited with stress, our data showing that BAPTA-AM treatment (Fig. 5D) in PC2 KD cells has no effect on cell viability compared to WT cells demonstrates the requirement for PC2-mediated Ca^2+^ signaling in the response to cell stress, whether this is through PC2’s intrinsic channel activity or its interactions with other channels.

Our study is limited by the variability of sample size throughout the different types of tissues and stress. For all conditions, we tested at least n = 3 samples to ensure that proper statistics could be performed during analyses. When possible, we maximized the sample size to increase the power of our study. However, the sample size of human and mouse tissues varied depending upon availability. Variables affecting this included limited access to human pathological samples with specific diagnoses, and the survival of mice undergoing stress procedures. Additionally, though our data point to a potential link between *PKD2* and *NFE2L2* expression, we do not presume to state that *NFE2L2* directly causes *PKD2* up-regulation based on our results. Rather, our data show that *PKD2* expression correlates with the activation of the ISR pathway due to its role as a stress response protein. The possibility remains that 1) increased Nrf2 may influence *PKD2* expression directly, 2) *NFE2L2* transcription may be increased due to altered signaling in cells with increased PC2, 3) *PKD2* and *NFE2L2* are up-regulated under similar stress conditions, but neither one directly affects the expression of the other, or 4) increased *PKD2* mRNA is independent of its transcription, but rather is a consequence of post-transcriptional changes such as mRNA stability. Consequently, if and how PC2 and Nrf2 influence one another’s expression, along with the exact mechanism of increased *PKD2* transcript will require further in-depth mechanistic studies.

Collectively, we describe a new functional role for PC2 as a stress-sensitive protein that is up-regulated in response to cellular insults in multiple disease states and tissue types. Moreover, loss of PC2 is sufficient to render cells hypersusceptible to stress-induced cell death, implicating altered stress response pathways in the development of polycystin-related pathologies. The ubiquitous expression of PC2 throughout the body raises the possibility that PC2 may contribute to disease states in other tissues where the ISR is induced or disrupted, and may provide a novel target to help ameliorate the progression of these diseases.

## Materials and Methods

### Reagents and antibodies

Anti-PC2 (sc-47734; for immunoblotting), anti-NFκB (sc-8008), and anti-IκBα (sc-1643) antibodies were purchased from Santa Cruz Biotechnology. Anti-PC2 antibody for immunofluorescent imaging was a kind gift from Dr. Stefan Somlo (Yale). Mouse anti-beta-Actin (#3700), rabbit anti-beta-Actin (#4970), anti-CHOP (#2895), anti-cleaved caspase-3 (#9664), and anti-GAPDH (#2118) antibodies were purchased from Cell Signaling Technology. Anti-4-Hydroxynonenal (ab48506), anti-eIF2α (ab50733), anti-phospho-eIF2α (ab32157) antibodies, and kainic acid (ab120100) were purchased from Abcam. Anti-VDAC (PA1-954A) antibody was purchased from Thermo Fisher Scientific. Hydrogen peroxide (H325-100) and BAPTA-AM (B1205) were purchased from Thermo Fisher Scientific. Tunicamycin (T7765), and Dimethyl sulfoxide (DMSO; W387520) were purchased from Sigma-Aldrich.

### Animal studies

The Yale University and University of Illinois at Urbana-Champaign Institutional Animal Care and Use Committees (IACUC) approved the animal housing conditions and the experimental procedures conducted in this study. All experiments were performed in accordance with relevant guidelines and regulations. C57BL/6 mice were kept under standard laboratory conditions with free access to food and water. Control mice were provided 2018S Teklad global rodent diet (Envigo). For diet-induced obesity, mice were fed TD.06414 Teklad diet containing 60% kcal/fat (Envigo) for 8 weeks-14 months, as described in the figure legends, beginning at 10 weeks of age. All animal experiments were performed in a blinded manner.

### Renal ischemia/reperfusion mouse model

Male 8- to 10-week-old C57BL/6 mice (National Institutes of Health/National Cancer Institute) were anesthetized with i.p. ketamine (100 mg/kg) and xylazine (10 mg/kg) then subjected to renal ischemia reperfusion (I/R) using a modified approach of that described previously^98^. Briefly, the right renal pedicle was isolated through a midline abdominal incision and clamped for 27 minutes using a nontraumatic microaneurysm clip (B-2, Fine Science Tools). The left kidney was removed and collected immediately before I/R of the right kidney (Sham, nephrectomized kidney). Mice were kept at 37 °C at all times during the procedure, and reperfusion of kidneys was confirmed after clamp release. Mice were given 1 ml normal saline intraperitoneally to prevent dehydration and were euthanized 72 hours after I/R. Kidney tissue was harvested for immunoblotting as described below.

### Glucose tolerance testing

At 18 weeks of age, mice were fasted for 16 hours before glucose tolerance testing. Glucose was administered via i.p. injection (1.5 g/kg body weight). Blood glucose measurements were taken immediately before injection and at 15, 30, 60, and 120 minutes following injection. Blood was collected using a lateral tail nick and glucose levels were measured using blood glucose strips and OneTouch glucometer (LifeScan).

### Kainic acid-induced acute seizure mouse model

At 8–10 weeks of age, male C57BL/6 J mice (Jackson Laboratory, Stock Number: 000664) received an i.p. injection of kainic acid (30 mg/kg body weight) or an equal volume of 0.9% saline as described^99^. Mice were returned to their cages and monitored for behavioral seizures every 10 minutes for 2 hours following injection. Behavioral seizures were scored according to a modified Racine scale as described previously^100,101^. At 24 hours post injection, mice were euthanized by CO_2_ inhalation and brains stored at −80 °C until use.

### Brain lysate preparation

The dissected brain regions per mouse were homogenized in ice-cold homogenization buffer (Solution A) containing (in mM): 320 sucrose, 1 NaHCO_3_, 1 MgCl_2_, 0.5 CaCl_2_, 0.4 HEPES (pH 7.4) and Halt protease inhibitors (Thermo Fisher Scientific) as previously described^99^. After centrifuging at 1400 x g for 10 minutes at 4 °C, the homogenate supernatant (S1) was separated from insoluble tissue and nuclear pellet (P1). The S1 fraction was then centrifuged at 13,800 x g for 10 min at 4 °C. The supernatant with cytosolic soluble proteins (S2) was removed, and the remaining pellet with transmembrane proteins and membrane-bound proteins (P2 membrane fraction) was resuspended in ice-cold solution B containing (in mM): 160 sucrose, 6 Tris-HCl, 0.5% Triton-X (pH 8.0) and Halt protease inhibitors. Pierce BCA assay (Thermo Fisher Scientific) was performed to normalize protein concentrations to 1 mg/ml in Solution B (pH 7.4). The S1, S2, and P2 fractions were stored at −80 °C until use.

### Western blot

Lysates of mouse kidneys, hearts, livers and human hearts were prepared by homogenization in radioimmunoprecipitation assay (RIPA) buffer containing SDS (Santa Cruz Biotechnology) with Halt protease and phosphatase inhibitor cocktail (Thermo Fisher). Lysates of mouse hippocampi were prepared as described above and the P2 fractions were run for western blotting. Cultured cells were lysed directly in M-PER mammalian protein extraction reagent (Thermo Fisher) supplemented with Halt protease and phosphatase inhibitor cocktail. Equal amounts of protein were loaded, and electrophoresis was performed in NuPAGE 4–12% gradient bis-tris polyacrylamide protein gels (Thermo Fisher). Proteins were transferred to PVDF membrane and blocked with 5% milk in phosphate-buffered saline with Tween-20 for 1 hour. Membranes were then incubated overnight with primary antibody at 4 °C. Blots were washed and incubated with secondary antibody for 1 hour at room temperature. After washing, the secondary antibody was visualized by Pierce ECL chemiluminescence reagents (Thermo Fisher) or using a LI-COR Odyssey imaging system (LI-COR Biosciences).

### Human tissue

Human kidney and liver tissues were identified and obtained through the Department of Pathology at the Yale School of Medicine. All experimental protocols and the use of these tissues for research were approved by the Yale Institutional Review Board. All methods were carried out in accordance with relevant guidelines and regulations. Informed consent was obtained from all subjects donating samples. Tissues were fixed with 4% paraformaldehyde at 4 °C overnight, embedded in paraffin, and 5 μm sections were cut for immunofluorescence. Following deparaffinization and rehydration, antigen retrieval was performed by incubating the sections in a steamer for 20 min in sodium citrate buffer (10 mM Tri-sodium citrate, 0.05% Tween-20, pH 6.0). Sections were then quenched of autofluorescence, blocked and permeabilized, and incubated with anti-PC2 (Santa Cruz Biotechnology), anti-lectin Dolichos biflorus agglutinin (DBA; Vector Laboratories), and anti-VDAC (Cell Signaling) antibodies. Tissues were mounted in ProLong Gold Antifade Mountant (Life Technologies) and imaged with 40× and 100× oil-immersion lenses on a Leica SP8 gated STED Microscope using Leica LAS X software (Heidelberg, Germany). To enable comparison among samples, the same laser power, gain and acquisition settings were used across all slides.

### Immunofluorescence

LLC-PK1 and mIMCD3 cells were immunostained as described previously^41^. Briefly, cells were plated on poly-L-lysine-coated glass coverslips and fixed with 4% paraformaldehyde for 10 minutes at 37 °C. After blocking in 2% BSA and 0.2% Triton X-100, cells were incubated in the same solution with primary antibody for active caspase 3 and PC2 overnight at 4 °C, then incubated in anti-rabbit AlexaFluor 488 (Life Technologies), phalloidin conjugated to tetramethylrhodamine (TRITC; Life Technologies), and anti-mouse AlexaFluor 633 (Life Technologies) for 1 hour before mounting with ProLong Gold antifade mounting reagent with DAPI (Life Technologies). Slides were imaged with a Leica TCS SP5 Confocal Laser Scanning Microscope.

To calculate PC2 and Cleaved Caspase 3 intensity, ImageJ (NIH) was used for image processing. All measurements of fluorescence intensity were calculated using images taken at 10X magnification to ensure unbiased and robust quantification. Individual cells or cell groups were selected as regions of interest (ROIs,) and the fluorescence intensity and area were calculated for each ROI. Intensity was divided by its corresponding area and averaged across each image. Five images were taken per condition and averaged to plot as Intensity/Area.

### Human heart RNA-Seq

RNA Sequencing was performed on left ventricular tissue samples from non-failing (NF; n = 8) human donor hearts, and from failing human hearts obtained from patients with ischemic cardiomyopathy (ICM; n = 8) or non-ischemic cardiomyopathy (NICM; n = 8) as described previously^48^. Data were analyzed using Qlucore Omics Explorer (Lund, Sweden). Normalized Reads Per Kilobase of transcript, per Million mapped reads (RPKM) values were log_2_ transformed and fold change calculated. Curated lists of genes associated with cellular responses to ER and oxidative stress were collected and cross-referenced from MetaCore (Clarivate Analytics) and Ingenuity Pathway Analysis (Ingenuity Systems, QIAGEN Bioinformatics). The genes were compiled and used to generate an expression heat map of differentially regulated (p < 0.05) ER and oxidative stress-related genes. Linear regression analysis was performed to show significant (p < 0.0001) correlation between *PKD2* and *NFE2L2* transcripts.

### iPSC culture and differentiation

Human induced pluripotent stem cells (iPSC) were acquired from the Coriell Institute (GM23338), as characterized previously^102^. iPSC colonies were maintained on growth-factor reduced Matrigel (Corning)-coated tissue culture plates in mTeSR1 medium (Stem Cell Technologies). iPSCs were differentiated into iPSC-derived cardiomyocytes (iPSC-CMs) following a sequential protocol of manipulating the WNT pathway^103^, using 12.5 μM CHIR99021 (Stem Cell Technologies) for 24 hours and 5 μM IWP4 (Tocris Bioscience) for 48 hours on day 3 after the induction of differentiation. Subsequently, the differentiating cultures were maintained in RPMI + B2-insulin until day 11 when spontaneous beating was observed. Media was then changed to RPMI + B27 + insulin for 2 days, followed by 4 days of metabolic selection using DMEM without glucose and 4 mM lactate^104^. iPSC-CMs were kept in RPMI + B27 + insulin until day 21 after the start of differentiation when they were dissociated and re-plated on a 12-well plate coated with 50 μg/ml fibronectin (bovine, Sigma-Aldrich)^105^. iPSC-CMs were cultured for a subsequent 2 days in RPMI + B27 + insulin before experiments were performed.

### Human glioblastoma project RNA-Seq

The open-source RNA-Seq dataset from The Allen Institute’s human glioblastoma project was used to interrogate the relationship between *PKD2* expression in human brain and integrated stress response genes. RNA-Seq was performed on different glioblastoma structures as described^62^ and the FPKM values log_2_ normalized before analysis with Qlucore Omics Explorer. Linear regression analysis was performed to show significant (p < 0.0001) correlation between *PKD2* and *NFE2L2* transcripts.

### Creation of stable knockdown cells

Short hairpin RNA (shRNA) against *Pkd2* was designed to be specific to the pig sequence. The previously described^41,106^ PC2 shRNA constructs were put into a psiRNA vector (InvivoGen, San Diego, CA), which was stably transfected into LLC-PK1 cells. Cells were exposed to G418 (2.5 mg/ml) as previously described^41,106^. LLC-PK1 cells were maintained in Dulbecco’s Modified Eagle Medium (DMEM) supplemented with 10% fetal bovine serum (FBS) and kept at 37 C in 5% CO_2_.

### *In vitro* stress treatment

Cells were plated on a 6-well plate (Corning) at a density of 400,000 cells per well. The following day, cells were either treated with the commonly used ER-stress inducer Tunicamycin (TM)^107^ or serum-starved for 24 hours. After treatment, the cells were lysed and collected as described above for Western Blot or stained for Immunofluorescence.

### Measurement of cellular stress

To assess the level of cellular stress in both *in vivo* and *in vitro* samples, we immunoblotted for several markers whose levels are changed in response to stress: 4-HNE, a reactive short-chain aldehyde formed during lipid peroxidation following oxidative stress^108^; CHOP, a protein involved in ER-stress mediated apoptosis^51^; eIF2α, whose phosphorylation (p-EIF2α) is the core event in the integrated stress response pathway^50^; cleaved caspase 3, a commonly used marker for apoptotic cells^109^.

### mRNA analysis

mRNA was extracted from animal tissue and cultured cells using a RNeasy RNA Isolation Kit (Qiagen, Hilden, Germany) and reverse-transcribed to complementary DNA (cDNA) using Multiscribe Reverse Transcriptase and random primers (Applied Biosystems, Foster City, CA). For real-time quantitative PCR, 20 ng of cDNA was used as transcript template in a reaction with Power SYBR Green Master Mix (Applied Biosystems) on a 7500 Fast Real-Time PCR system (Applied Biosystems). Fold change in *Pkd2* (Fwd 5′—CAGAGGGGCTGCTACAGTTT—3′; Rev 5′—TTGAAGAGCTTAATCCAGACCA—3′) and Nfe2l2 (Fwd 5′—AGATGACCATGAGTCGCTTGC—3′; Rev 5′—CCTGATGAGGGGCAGTGAAG—3′) mRNA transcript levels compared to control samples were determined using the 2^−(ΔΔCT)^ method. *Actin* (Fwd 5′—GTGACGTTGACATCCGTAAAGA—3′; Rev 5′—GCCGGACTCATCGTACTCC—3′) or *Gapdh* (Fwd 5′—AGGTCGGTGTGAACGGATTTG—3′; Rev 5′—GGGGTCGTTGATGGCAACA—3′) were used as internal control.

### CellTiter-Glo assay

Cells were plated at a density of 1000 cells per well on a 96-well plate (Corning) and treated the following day. After 24 hours of treatment, CellTiter-Glo Luminescent Cell Viability assays were carried out according to the manufacturer’s instructions (Promega Corporation). Experiments were performed in triplicates with 10 replicates per experiment.

### Statistical analysis

For cell-based experiments, data were calculated from at least three separate preparations. For animal-based experiments, data were calculated from at least three biological replicates. Where appropriate, one-way analysis of variance (ANOVA) with multiple comparisons or non-parametric Mann Whitney U test was applied. Data are presented as (1) mean ± SD, or (2) median with range, as stated in the figure legend. In all experiments, p < 0.05 was considered statistically significant. *p < 0.05, **p < 0.01, ***p < 0.001, and ****p < 0.0001.

## Supplementary information

Supplementary Information.

## Data Availability

The data generated, used, and/or analyzed in the current study are available from the corresponding author upon reasonable request.
