# Supplementary material for: Polycystin 2 is increased in disease to protect against stress-induced cell death
Source: Sci Rep. 2020 Jan 15;10:386. doi: 10.1038/s41598-019-57286-x (PMC6962458; doi:10.1038/s41598-019-57286-x)
Supplement: Supplementary file 1 — Supplementary Information. [file 41598_2019_57286_MOESM1_ESM.pdf]

Title: Polycystin 2 is increased in disease to protect against stress-induced cell death

Authors: Allison L. Brill[1], Tom T. Fischer[2,3], Jennifer M. Walters[4,5], Arnaud Marlier[6], Lorenzo R. Sewanan[7], Parker C. Wilson[8,†], Eric K. Johnson[9], Gilbert Moeckel[8], Lloyd G. Cantley[6], Stuart G. Campbell[7], Jeanne M. Nerbonne[9,10], Hee Jung Chung[4,5], Marie E. Robert[8], and Barbara E. Ehrlich[1,2,*]

Affiliations:

Departments of [1]Cellular and Molecular Physiology, [2]Pharmacology, [6]Internal Medicine, [7]Biomedical Engineering, and [8]Pathology, Yale University, New Haven, CT 06510, United States of America

[3]Institute of Pharmacology, Heidelberg University, Heidelberg, Germany

[4]Department of Molecular and Integrative Physiology, [5]Neuroscience Program, University of Illinois at Urbana-Champaign, Urbana, IL 61801, United States of America

Departments of [9]Medicine, Cardiovascular Division, and [10]Developmental Biology, Washington University School of Medicine, St. Louis, MO 63110, United States of America

†Current address: Department of Pathology and Immunology, Washington University in St. Louis, St. Louis, MO, 63110, United States of America

*Corresponding author

E-mail: barbara.ehrlich@yale.edu (BEE)

Department of Pharmacology, 333 Cedar Street

New Haven, CT 06510

SUPPLEMENTARY MATERIALS

Supplementary methods

Micro-osmotic pump implant

Micro-osmotic pump implants were performed as described previously[35]. All mouse studies were performed with male mice, using wild- littermates as controls. The animals were 5 weeks old at the beginning of the experimental procedures. Mice received general anesthesia consisting of a vapor mixture of 2.5% sevoflurane, 5% nitrous oxide and 95% oxygen. Additionally, 0.1 ml of 1% lidocaine was used as a local anesthetic and injected subcutaneously in the interscapular area. Micro-osmotic pumps (ALZET) were filled with 25 µg × g body weight of D/L-hydrochloride-isoproterenol (diluted in 0.9% phosphate-buffered saline and 0.5 mmol/L ascorbic acid) or only the diluent. An aseptic incision was made, and the micro-osmotic pump was subcutaneously implanted in the back of the mice. The drinking water was supplemented with 1 mg/ml ibuprofen for the following 48 hours after the micro-osmotic pump implantation. The pump content was delivered constantly over a period of seven days at a rate of 25 µg/g body weight/day. Left ventricles (LV) were isolated from anesthetized mice and flash-frozen in liquid nitrogen for western blot analysis.

Generation of PKD2 knockout mIMCD3 cell line

Cas9 mutant control (Cas9 D10A) and PKD2 bi-allelic knockout (PC2 KO) mIMCD3 cells were generated as described previously[11]. Briefly, sgRNA against exons 2 and 3 of *PKD2* were cloned into a pGL3-U6-sgRNA-PGK-hygromycin plasmid, which was obtained from the modified pGL3-U6-sgRNA-PGK-puromycin plasmid (Plasmid #51133, Addgene). Cas9 D10A lentivirus was infected into mIMCD3 cell lines and the infected cells were selected with puromycin to obtain stable Cas9 D10 mIMCD3 control cells. pGL3-U6-sgRNA-PGK-hygromycin with *PKD2*-specific sgRNAs were transfected into the control

cells, and individual cells were transferred into 96-well plates after selection with hygromycin and puromycin to generate PC2 knock-out (PC2 KO) cells. Cas9 D10A and PC2 KO mIMCD3 cells were maintained in DMEM/F-12 50:50 supplemented with 10% fetal bovine serum and kept at 37°C in 5% CO_2.

SUPPLEMENTARY FIGURES

Fig S1. Kidneys with AKI have increased cellular stress and PC2 levels

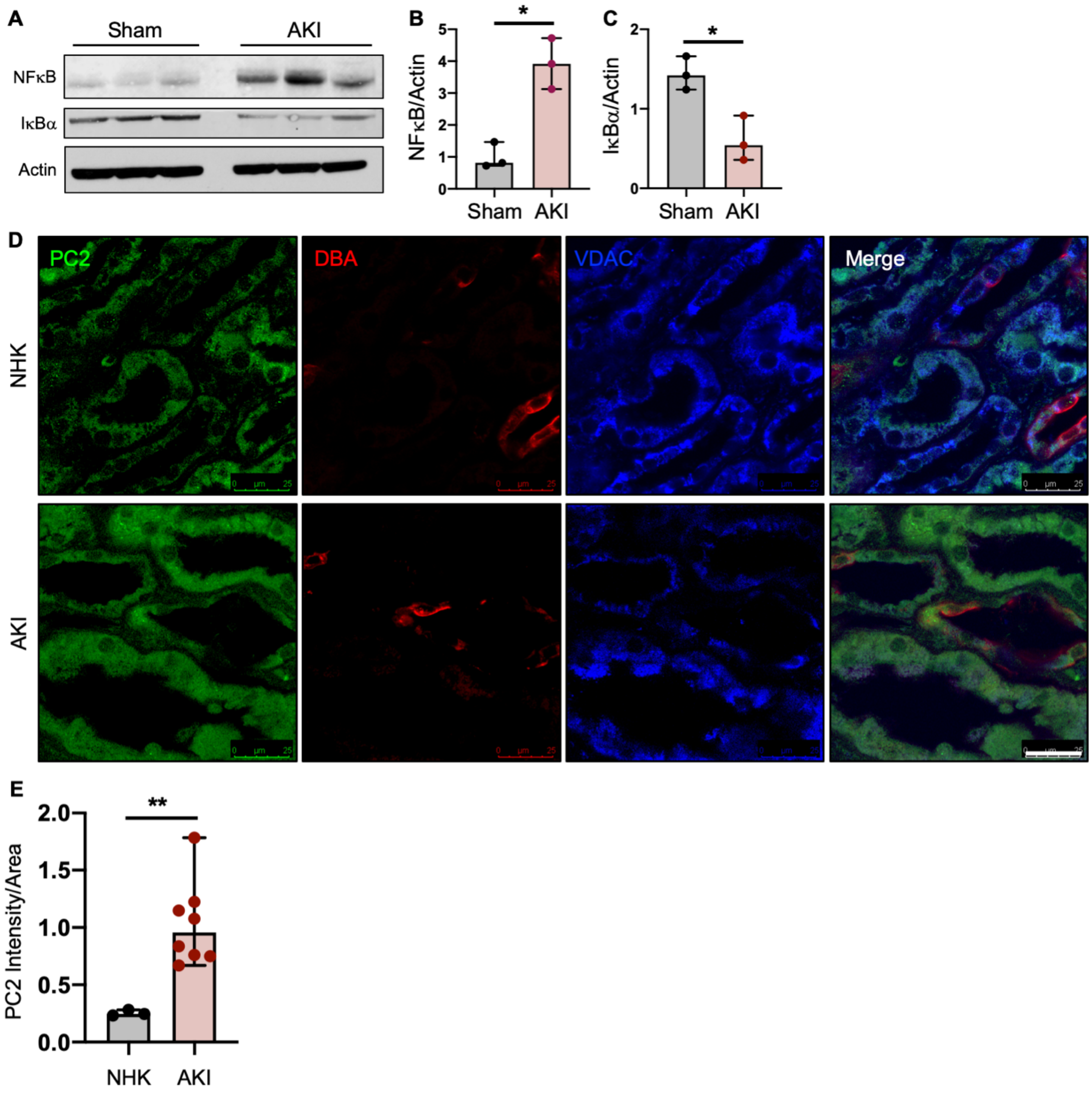

(**A**) Kidneys from control (sham-operated) or AKI-afflicted mice were immunoblotted for NFκB and IκBα. Each lane represents one biological replicate; n=3. (**B**,**C**) Quantification of NFκB and IkBα abundance in AKI versus sham kidneys normalized to actin. *p<0.05 as determined by Mann Whitney U

test. Data presented as median with range. (**D**) Normal human kidneys (NHK) or kidneys diagnosed with acute tubular injury (AKI) were stained for PC2 (green), DBA (red), and VDAC (blue). Scale bar, 25 μm. (**E**) PC2 intensity normalized to cell area was quantified in NHK and AKI human kidneys. **$p<0.01$ as determined Mann Whitney U test. Quantification is of 5 images per sample; Sample number NHK n=3, AKI n=8.

Fig S2. Livers with NAFLD have increased cellular stress and PC2 levels

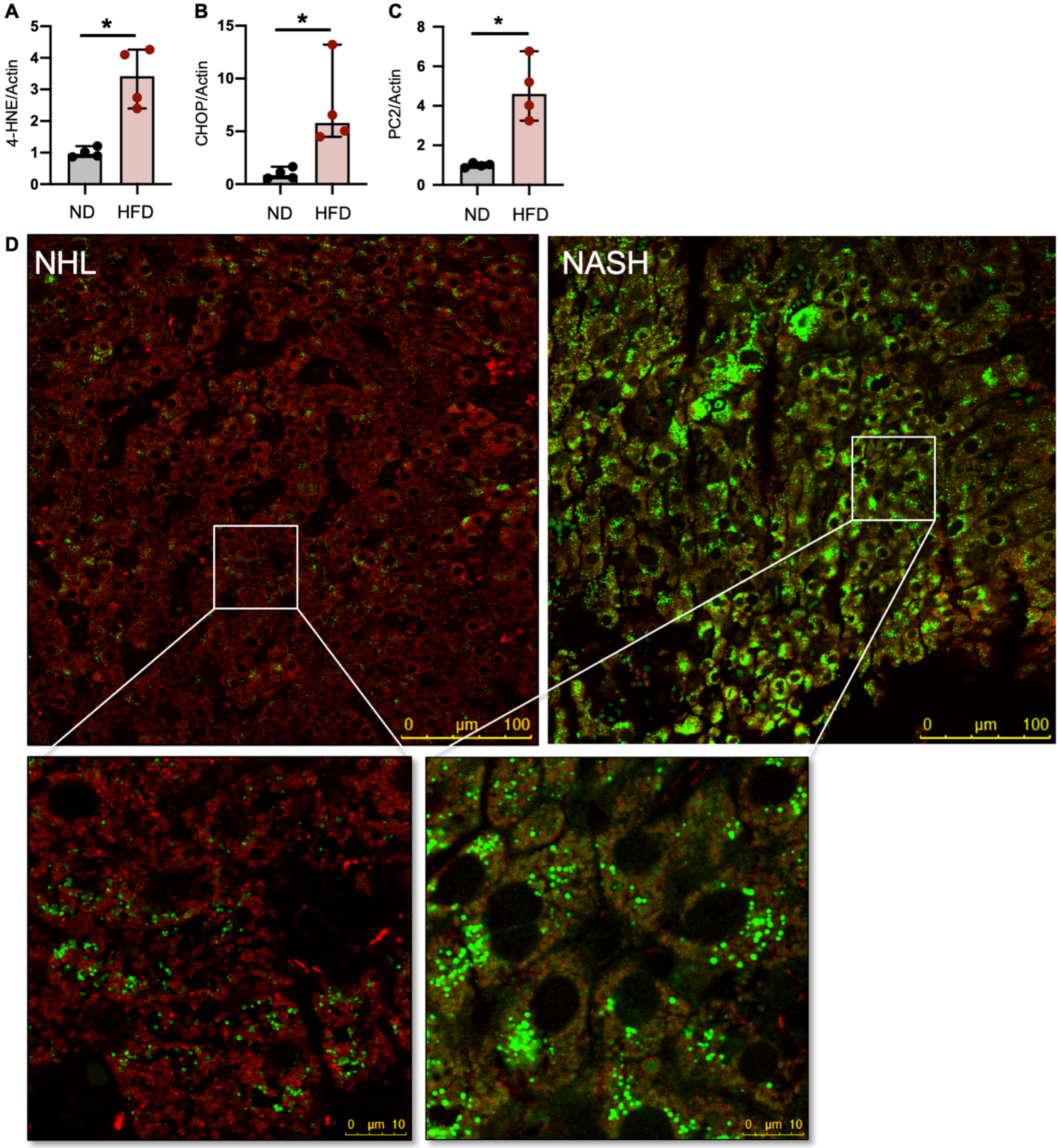

(**A**-**C**) Quantification of 4-HNE, CHOP, and PC2 abundance in livers from mice fed ND versus HFD, normalized to actin. *p<0.05 as determined by Mann Whitney U test. Data presented as median with range.

Sample size n=4 per group. (**D**) Normal human livers (NHL) and human livers with non-alcoholic steatohepatitis (NASH) were immunostained for PC2 (green) and VDAC (red). Scale bar, 100 μm (top); scale bar, 10 μm (bottom).

Fig S3. PC2 is increased in stressed hearts and correlates with NFE2L2 expression

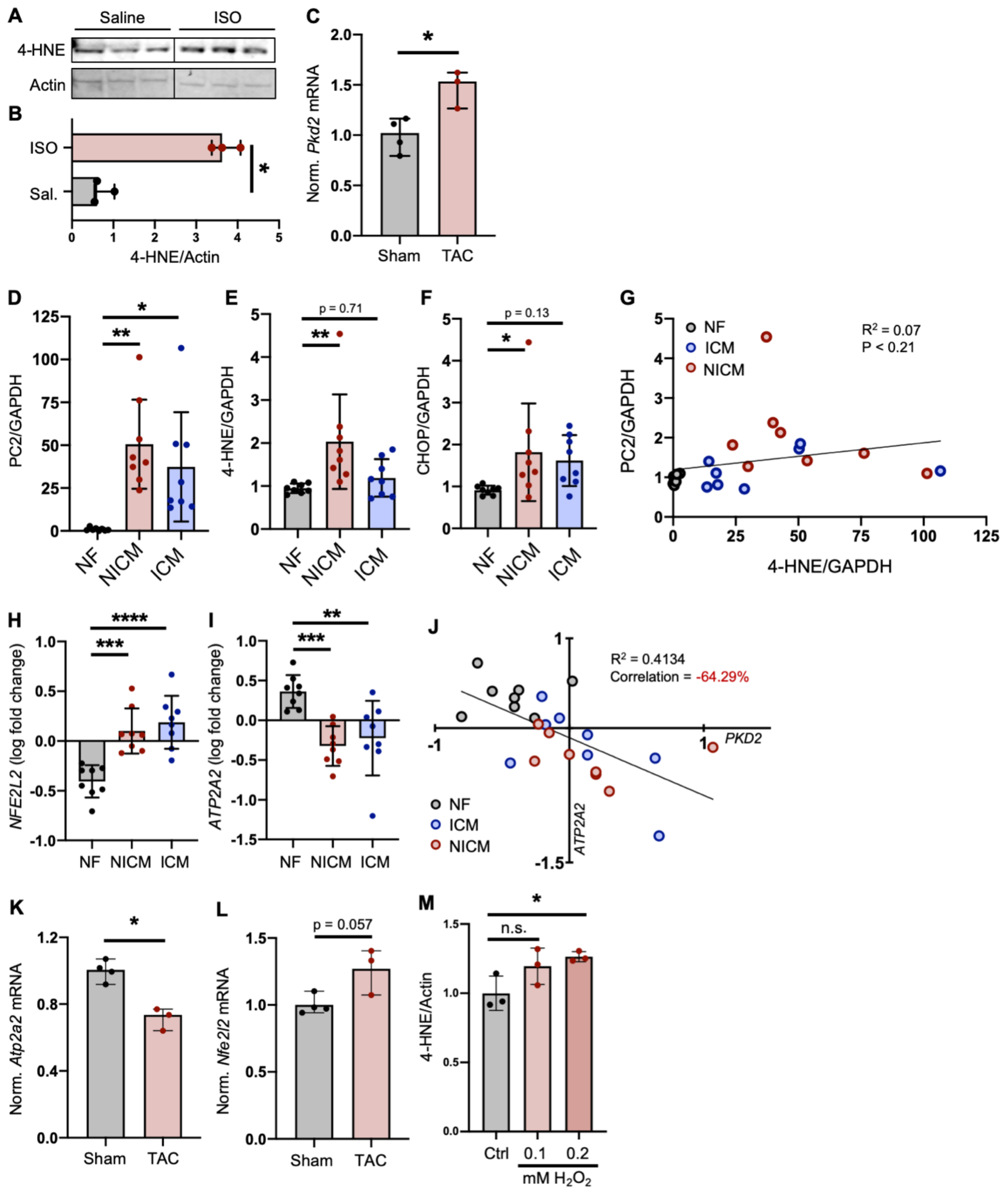

(**A**) Left ventricles of hearts from mice treated with saline or ISO for 7 days were immunoblotted for 4-HNE. Each lane represents one biological replicate; n = 3. Rows were cut to exclude samples irrelevant

to this study. Full-length blots shown in Fig. S10. (**B**) Quantification of 4-HNE abundance in the hearts of saline (Sal.) versus ISO-treated mice, normalized to actin. *$p<0.05$ as determined by Mann Whitney U test. Data presented as median with range. (**C**) Fold change of *Pkd2* mRNA in the left ventricles of Sham versus stressed (TAC) mice. *$p<0.05$ as determined by Mann Whitney U test. Data presented as median with range. (**D-F**) Quantification of PC2, 4-HNE, and CHOP abundance in NF versus NICM and ICM human hearts, normalized to GAPDH. *$p<0.05$; **$p<0.01$ as determined by one-way ANOVA. Data presented as mean $\pm$ SD. (**G**) Normalized protein expression of PC2 was plotted against each sample's corresponding normalized 4-HNE expression. (**H**,**I**) Log fold change of the ISR genes *NFE2L2* and *ATP2A2* in NF versus ICM and NICM human hearts. **$p<0.01$; ***$p<0.001$; ****$p<0.0001$ as determined by one-way ANOVA. Data presented as mean $\pm$ SD. (**J**) *ATP2A2* does not correlate with *PKD2* expression at the cutoff of 70% correlation or higher. (**K**,**L**) Fold change of *Atp2a2* and *Nfe2l2* in the left ventricles of Sham versus stressed (TAC) mice. *$p<0.05$ as determined by Mann Whitney U test. Data presented as median with range. (**M**) Quantification of 4-HNE abundance in human iPSC-CMs treated with increasing concentrations of H_2O_2. *$p<0.05$ as determined by one-way ANOVA.

Fig S4. PC2 up-regulation correlates with differential ISR pathway activation in stressed brains

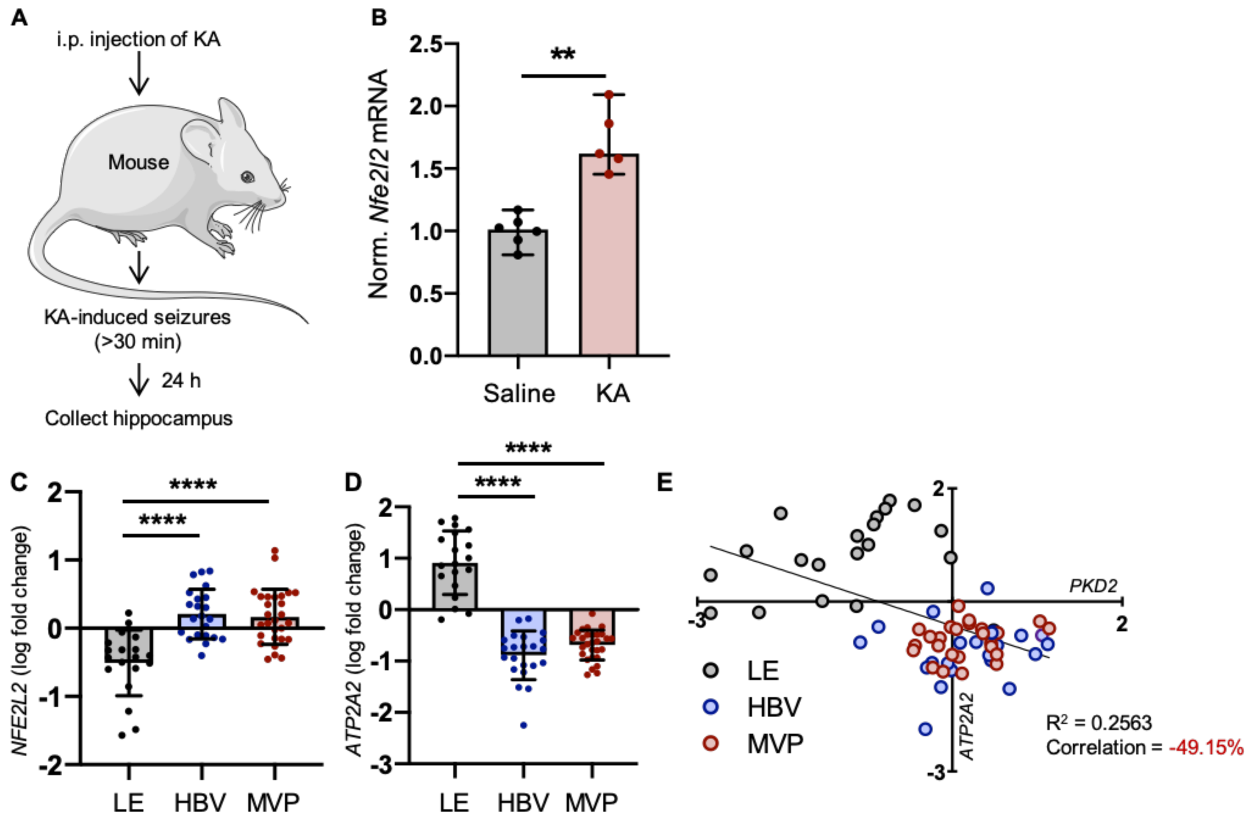

(**A**) Schematic of the experimental procedure using kainic acid to induce epileptic seizures in mice. (**B**) Fold change of *Nfe2l2* mRNA in hippocampi of saline- or KA-treated mice. **p<0.01 as determined by Mann Whitney U test. Data presented as median with range. Sample size Saline n=6, KA n=5. (**C,D**) Log fold change of the ISR genes *NFE2L2* and *ATP2A2* in LE versus HBV and MVP tumor samples from human glioblastoma. ****p<0.0001 as determined one-way ANOVA. (**E**) *ATP2A2* does not correlate with *PKD2* expression at the cutoff of 70% correlation or higher.

Fig S5. PC2 protects against stress-induced apoptosis

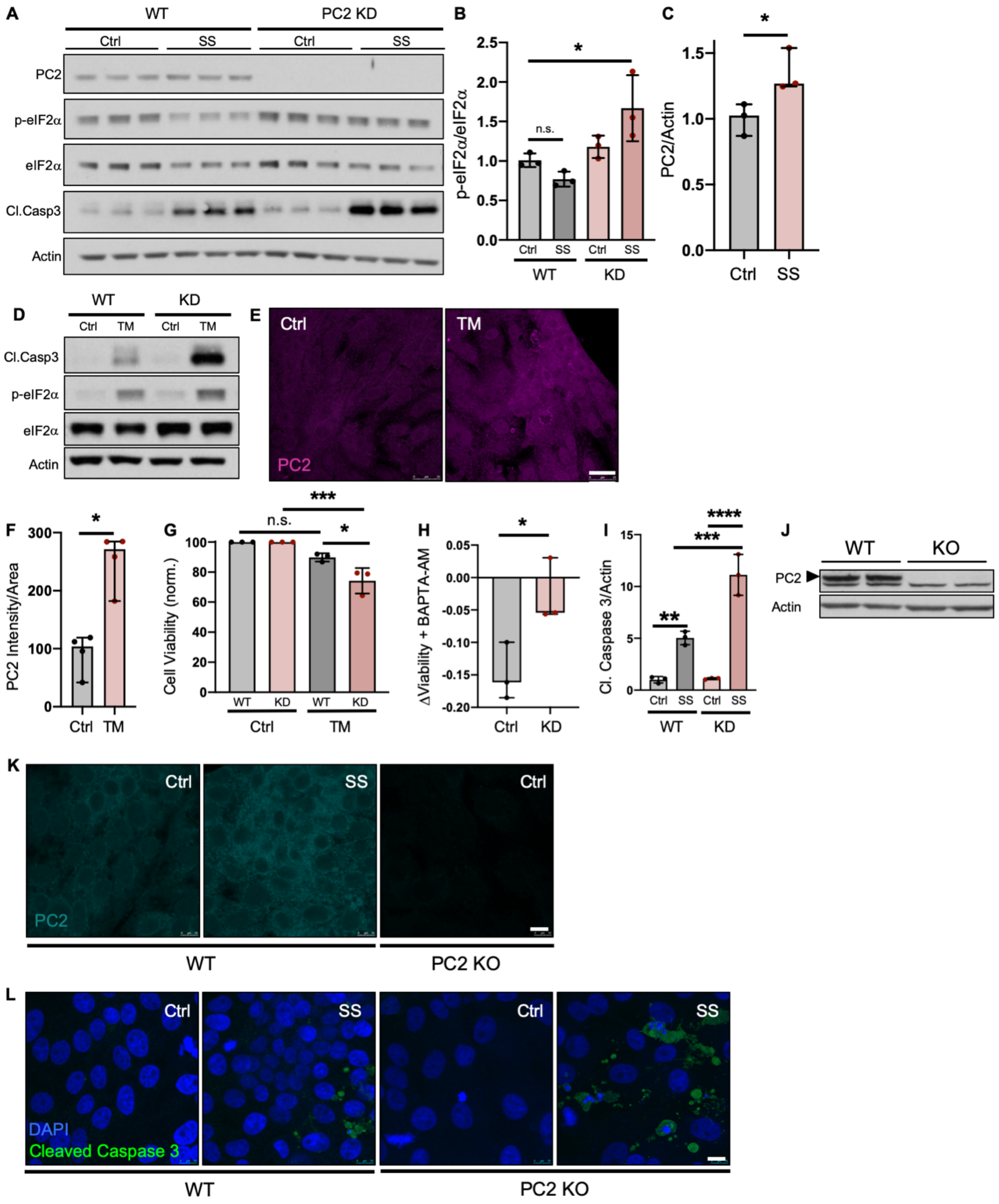

(**A**) WT and PC2 KD LLC-PK1 cells were grown in normal culture medium (Ctrl) or base medium without FBS (SS) for 24 hours and immunoblotted for PC2 and the key ISR pathway component, phospho-eIF2α (p-eIF2α). Each lane represents one biological replicate; sample size n=3 per group. Full-length blots shown in Fig. S12. (**B**) Quantification of p-eIF2α abundance in WT and PC2 KD cells under Ctrl and SS conditions, normalized to total eIF2α. *p<0.05 as determined by one-way ANOVA. Data presented as mean ± SD. (**C**) Quantification of PC2 abundance in WT and PC2 KD under Ctrl and SS conditions, normalized to actin. *p<0.05 as determined by Mann Whitney U test. Data presented as median with range. (**D**) Western blot analysis of active eIF2α (p-eIF2α/eIF2α) and caspase 3 (Cl.Casp3) in WT and PC2 KD cells treated with DMSO (Ctrl) or 3 μg/ml TM for 24 hours confirmed the induction of ER stress in LLC-PK1 cells. Full-length blots shown in Fig. S13. (**E**) LLC-PK1 cells were grown in the presence of DMSO (Ctrl) or 3 μg/ml TM for 24 hours, then stained for PC2. Images shown are representative of one Ctrl and one TM-treated sample from a total of 3 biological samples. Scale bar, 25 μm. (**F**) PC2 fluorescence per cell area was quantified in Ctrl and TM-treated LLC-PK1 cells. *p<0.05 as determined by Mann Whitney U test. Data presented as median with range. (**G**) Viability of WT and PC2 KD cells grown in the presence of DMSO (Ctrl) or 3 μg/ml TM for 24 hours was tested via CellTiter-Glo assay. *p<0.05 and ***p<0.001 as determined by one-way ANOVA. Data presented as mean ± SD. (**H**) Change in cell viability in serum-starved (SS) WT and PC2 KD cells with the addition of 100 nM BAPTA-AM for 24 hours. *p<0.05 as determined by Mann Whitney U test. Data presented as median with range. (**I**) Quantification of cleaved caspase 3 abundance in WT and PC2 KD cells under Ctrl and SS conditions, normalized to actin (as measured by immunoblot in [A]). **p<0.01, ***p<0.001, and ****p<0.0001 as determined by one-way ANOVA. Data presented as mean ± SD. (**J**) WT and PC2 KO mIMCD-3 cells were immunoblotted for PC2. Each lane represents one biological replicate. The top band (arrow) shows PC2 and the bottom band non-specific binding. Full-length blots shown in Fig. S14. (**K**) mIMCD-3 cells were grown in normal

culture medium (Ctrl) or serum starved (SS) for 24 hours, then stained for PC2. Images shown are representative of one image from a total of 3 biological samples. The absence of PC2 immunofluorescence was confirmed in PC2 KO cells. Scale bar, 10 μm. (**L**) Ctrl and 24h SS WT and PC2 KO mIMCD-3 cells were stained for DAPI (blue) and cleaved caspase 3 (green). Images shown are representative of 3 biological samples per group. Scale bar, 10 μm.

Fig S6. Full-length blots for 4-HNE, PC2, Actin, NFκB, and IκBα in mouse kidneys

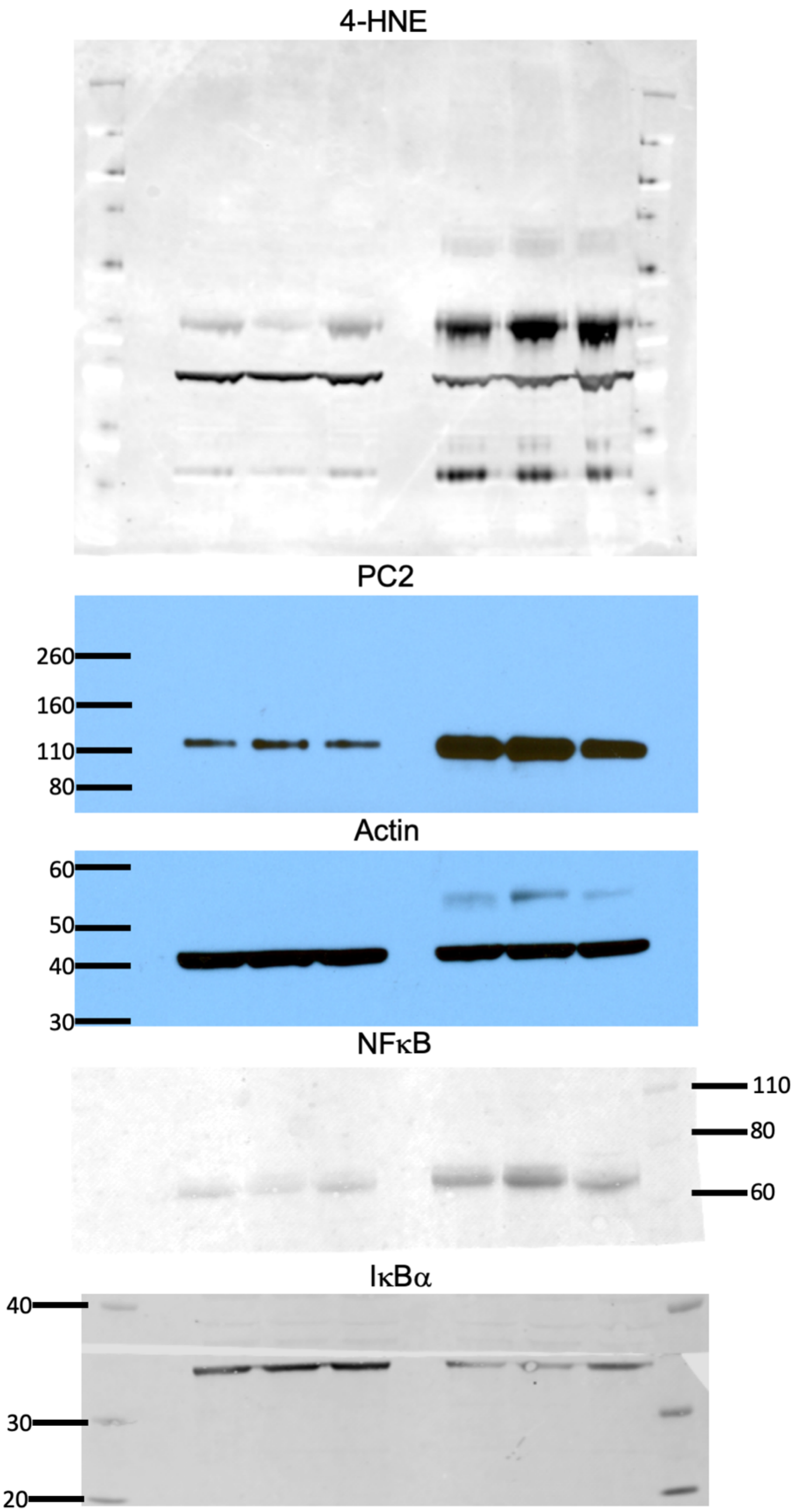

Fig S7. Full-length blots for PC2, 4-HNE, CHOP, and Actin in mouse livers

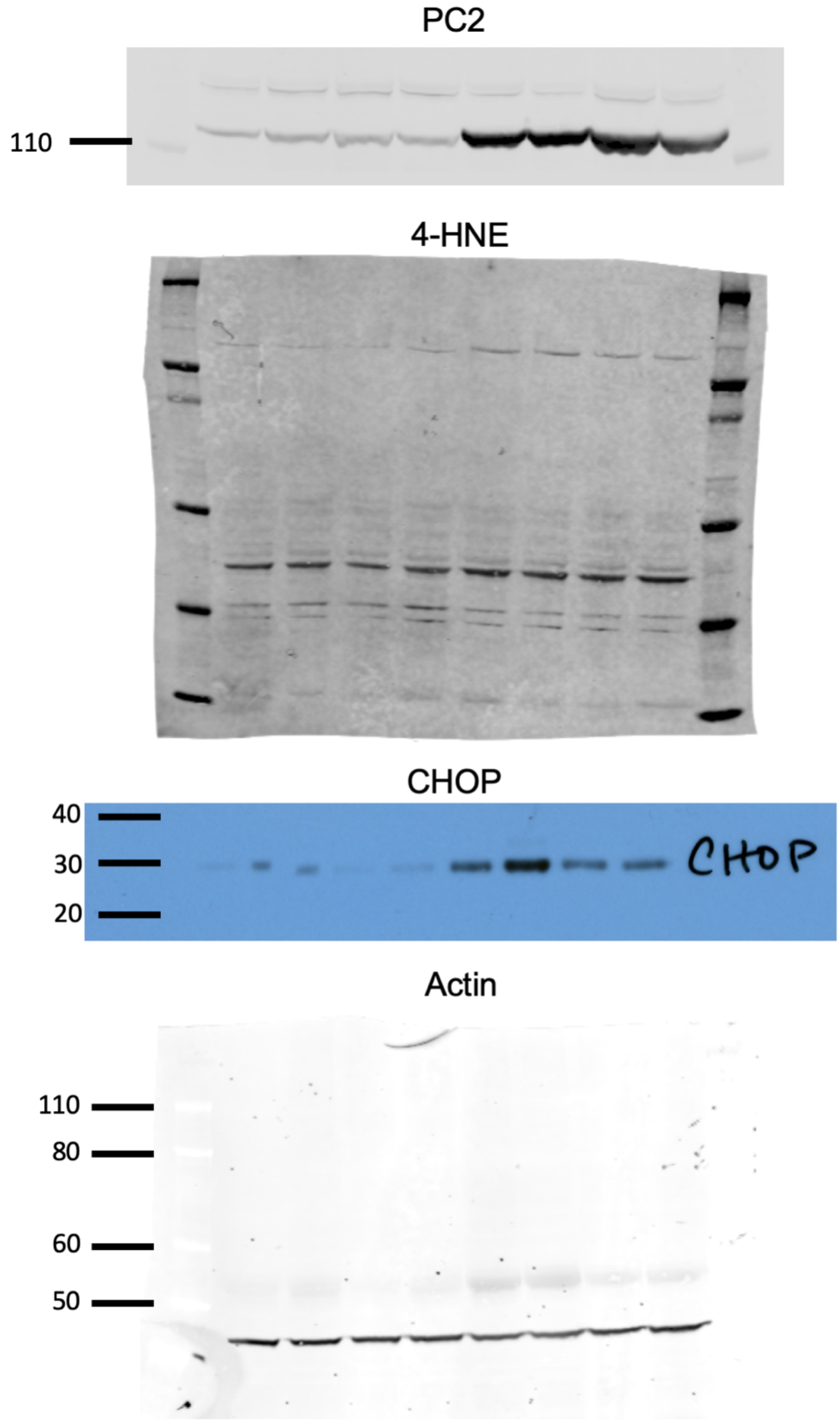

Fig S8. Full-length blots for PC2, 4-HNE, CHOP, and GAPDH in human hearts

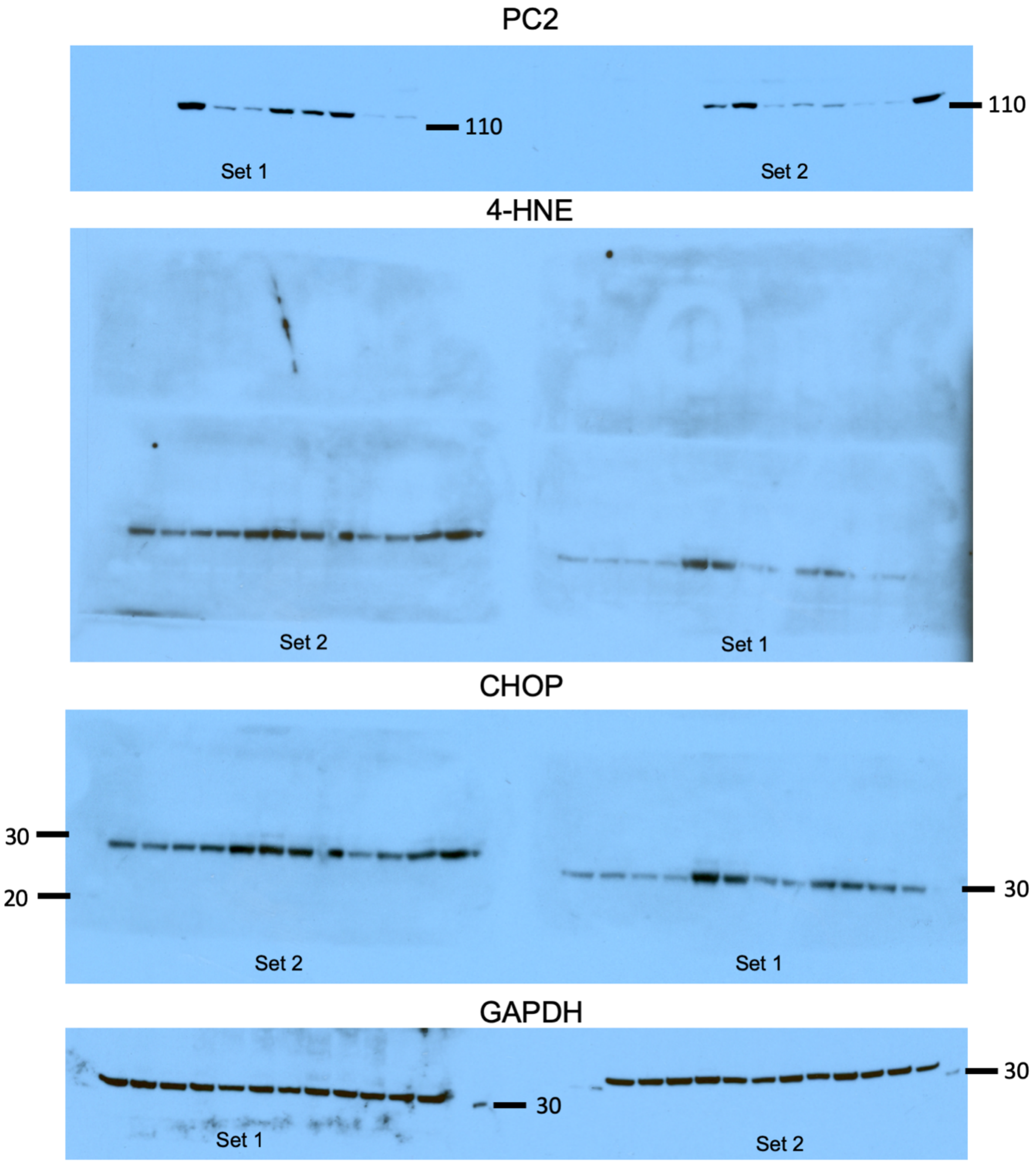

Fig S9. Full-length blots for PC2, 4-HNE, and Actin in iPSC-CMs

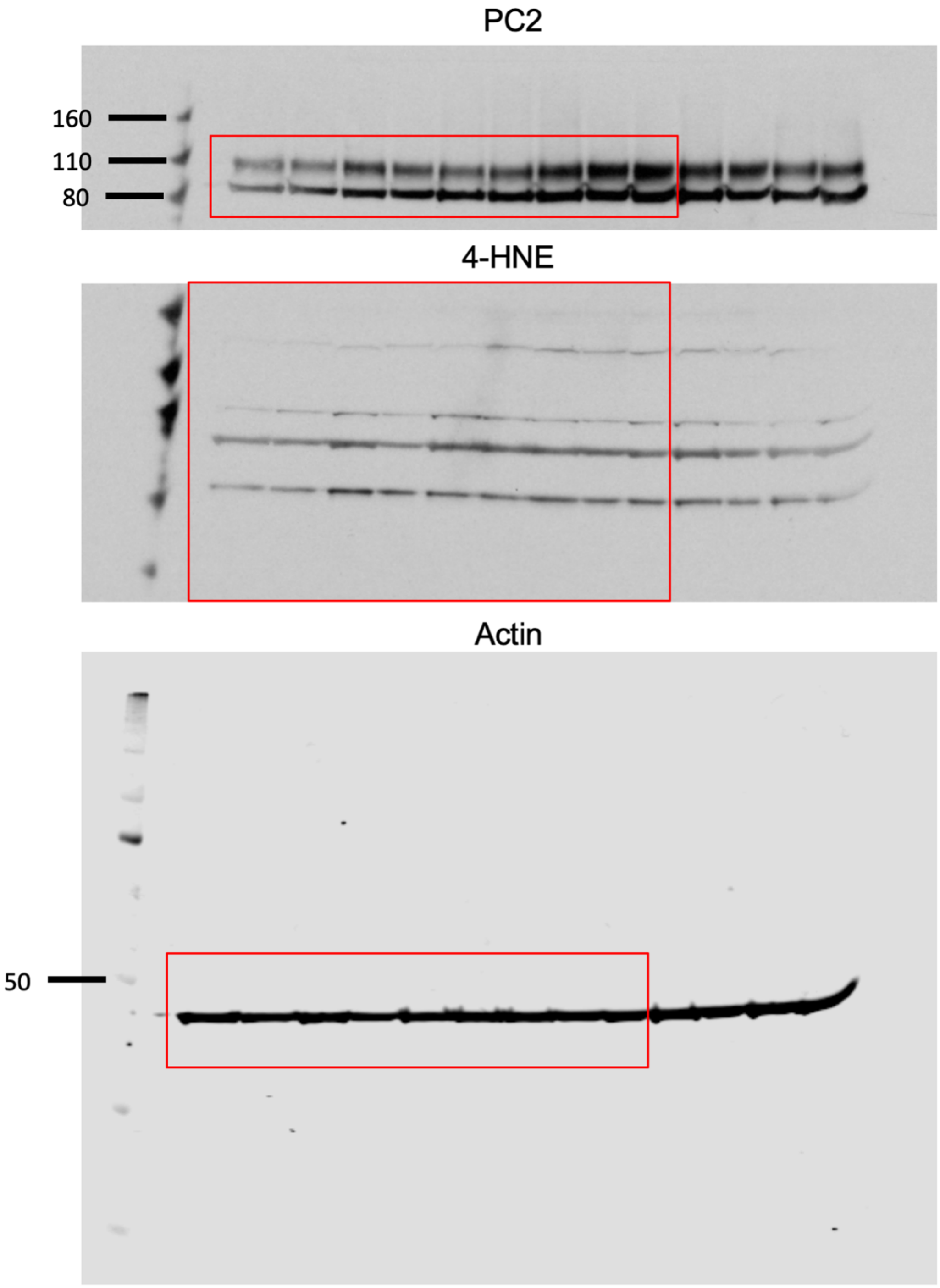

Fig S10. Full-length blots for 4-HNE and Actin in mouse hearts

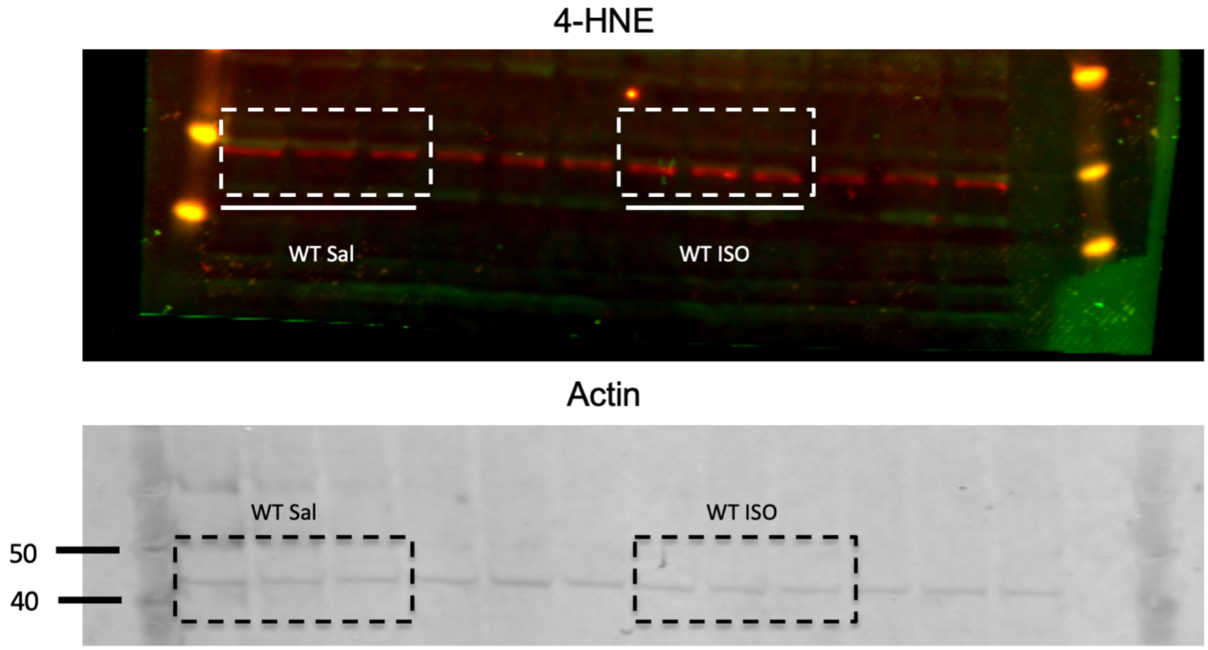

Fig S11. Full-length blots for PC2 and Actin in mouse hippocampi

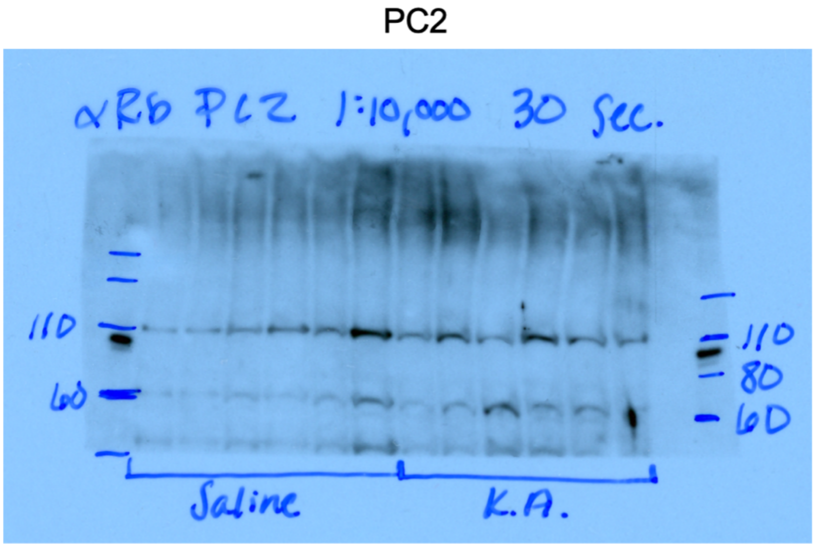

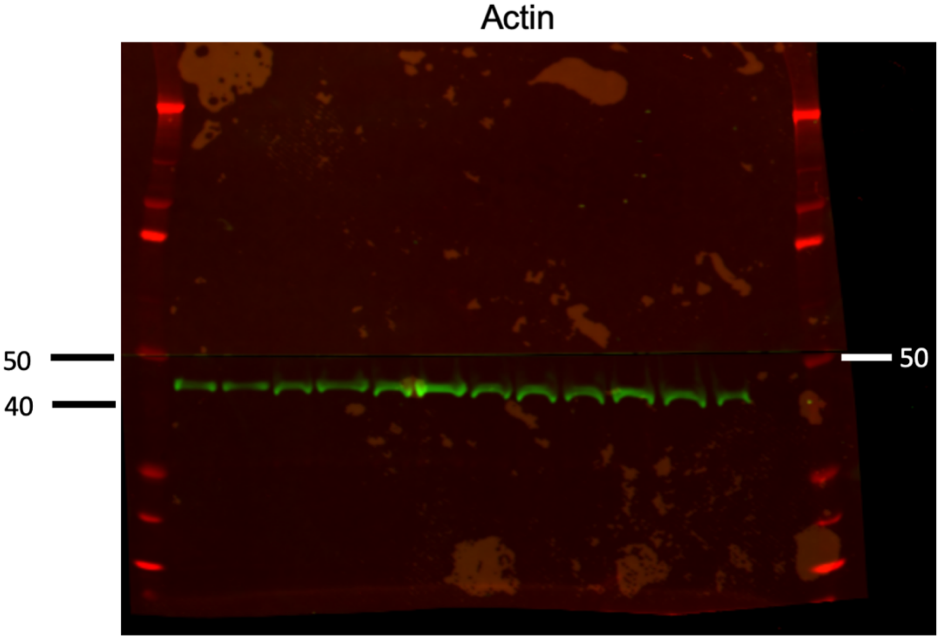

Fig S12. Full-length blots for PC2, p-eIF2α, eIF2α, Cleaved Caspase-3, and Actin in serum-starved LLC-PK1 cells

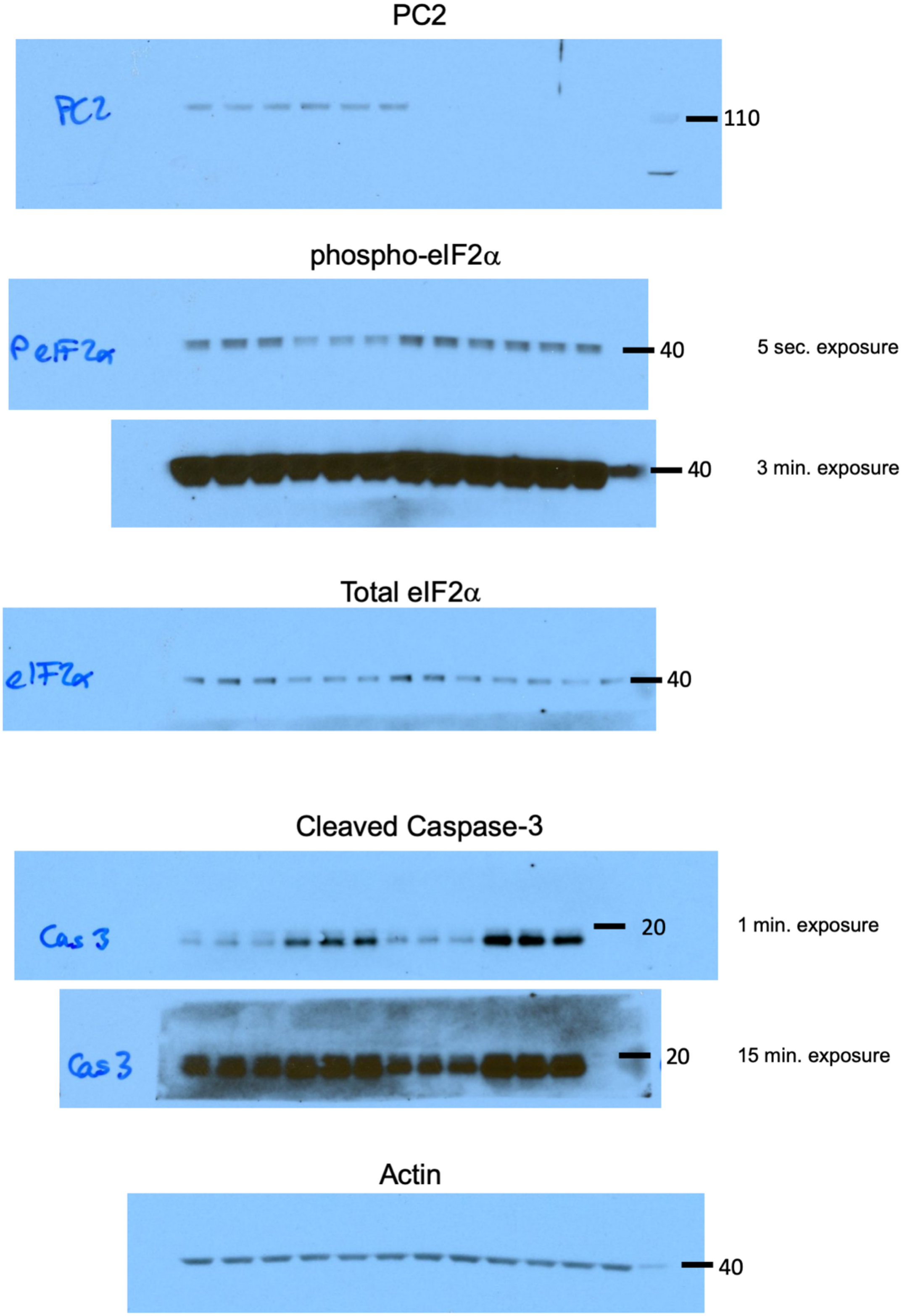

Fig S13. Full-length blots for Cleaved Caspase-3, p-eIF2α, eIF2α, and Actin in Tunicamycin-treated LLC-PK1 cells

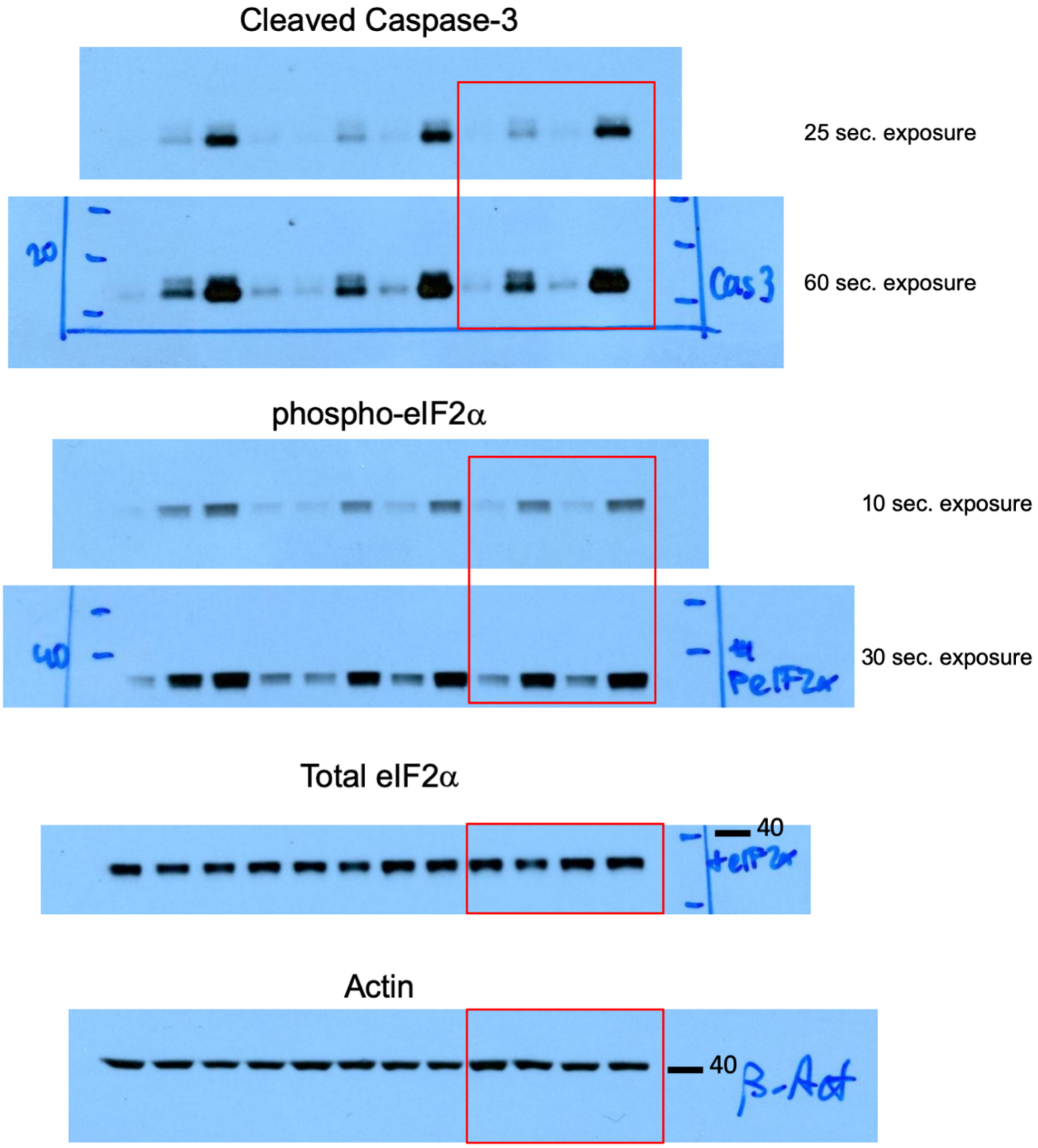

Fig S14. Full-length blots for PC2 and Actin in mIMCD3 cells

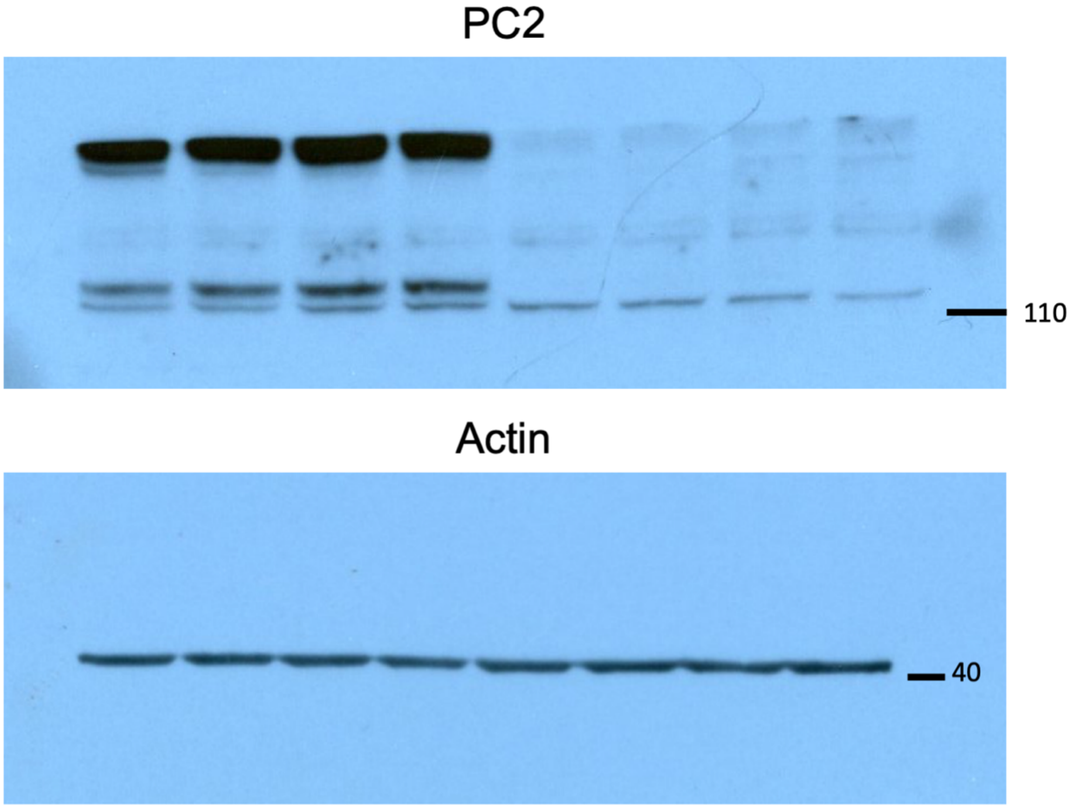

Supplementary Table 1

Case #	Sex	Age	Diagnosis
S15-26971 (Block 1-13)	F	60	Normal' kidney tissue from radical tumor nephrectomy specimens
S16-4540 (Block 1-8)	F	64	Normal' kidney tissue from radical tumor nephrectomy specimens
S16-7194 (Block 1-9)	M	64	Normal' kidney tissue from radical tumor nephrectomy specimens
S16-8062 (Block 1-1)	F	20	Acute Tubular Injury
S16-11651 (Block 1-1)	F	32	Acute Tubular Injury
S16-17174 (Block 1-1)	M	65	Acute Tubular Injury
S16-15208 (Block 1-1)	F	48	Acute Tubular Injury
S16-24822 (Block 1-1)	M	77	Acute Tubular Injury
S16-26779 (Block 1-1)	M	73	Acute Tubular Injury
S16-33449 (Block 1-1)	F	36	Acute Tubular Injury
S16-33546 (Block 1-1)	F	43	Acute Tubular Injury

Supplementary Table 2

Case #	Sex	Age	Stage	Diagnosis
S13-27339	M	68	4/4	True NASH; mild steatosis with mild steatohepatitis
S09-29159	M	60	4/4	True NASH; mild steatosis with minimal steatohepatitis
S13-10437	F	79	4/4	NASH; mild steatosis with minimal steatohepatitis
S09-16896	M	61	2/4	NASH; moderate steatosis with moderate steatohepatitis
S10-25573	F	56	4/4	True NASH; moderate steatosis with moderate steatohepatitis
S13-18494	M	72	3-4/4	NASH; moderate steatosis with moderate steatohepatitis
S14-29900	F	72	2/4	NASH; moderate steatosis with moderate steatohepatitis
S16-9546	M	58	4/4	True NASH; mild steatosis with mild steatohepatitis
S15-23977	M	62	0/4	NASH; mild steatosis without steatohepatitis

Supplementary Table 3

Sample #	Sex	Race	Age (yr)	Devices				Surgery	Comorbidities				Pre-VAD		Post-VAD	Pre-VAD Echo		Post-VAD Echo		Medication								Arrhythmia History	
				ICD	BiV	LVAD	Months of VAD		MI/CAD	Valve Disease	DM	History of Smoking	PAH	PAP	PAP	LVEDD (cm)	EF (%)	LVEDD (cm)	EF (%)	ASA	Statin	Amio	Mexil	β-Block	Inotrope	ACE-I	Coumadin	Atrial	Ventricular
ICM1	M	B	54	X	X	X	5.6	CABG	Y	severe PR	Y	Y	Y	53/30	34/15	6.8	20-25	6	<25	X	X	X		X		X	X		VF
ICM2	M	W	55	X	X	X	3.7	CABG	Y	N	N	Y	Y	66/32	41/24	6.6	21	6.2	10	X	X			X			X		
ICM3	M	W	63	X	X	X	16.9	CABG	Y	N	N	Y	Y	27/19	21/10		<15	5.7	<20	X	X	X					X	AF	
ICM4	M	W	65	X		X	18.9	CABG	Y	severe MR	N	Y	Y	66/24	25/18	6	22	5.2	<20	X	X	X						AF	
ICM5	M	W	65			X	4.8		Y	mild	Y	N	Y	58/27	29/22	6.6	17	4.9	18	X	X			X			X	AF	
ICM6	M	W	67	X		X	14.1	stents, biVADs	Y	AR, TR, MR	Y	Y	Y	50/26	23/11	7.9	10	7.7	<20	X	X			X	X	X	X		
ICM7	M	B	62	X		X	4.6		Y	severe TR/MR	Y	N	Y	39/25	ND	8	10	2.9	10	X	X	X		X				AF	
ICM8	M	W	66			X	6.8	AV repair	Y	mild MR,AR	N	Y	Y	76/38	ND	7.4	24	6.3	35	X	X	X		X	X	X	X		VT
NICM1	F	W	65	X	X	X	4.2	myectomy	N	mild	N	Y	Y	40/26	27/17		15	4.0	<20			X					X	AF	
NICM2	M	W	48	X	X	X	3.9		N	mild	N	N	Y	47/31	27/19	7.6	10-15	7.4	<20	X		X	X	X	X		X	AF	VT
NICM3	F	W	35			X	5.6		N	mild	Y	Y	Y	35/19	21/12	6.4	20-25	5.1	20	X				X			X		
NICM4	M	W	53	X	X	X	9.8		N	MR	N	N	Y	37/17	29/17	7	15	7.8	5	X	X	X		X			X		VF
NICM5	M	W	58	X	X	X	5.6		N	severe MR	N	Y	Y	40/21	14/5	8.1	12		<20	X	X			X			X	SVT	VT
NICM6	M	W	60	X		X	15.7		N	N	Y	Y	Y	36/18	33/18		15	6.3	<35	X	X	X		X			X		NSVT
NICM7	M	B	55	X		X	19.5		N	N	Y	N	Y	64/36	41/23	6.4	13	5.4	<20	X	X	X			X		X	AF	
NICM8	M	B	64	X	X	X	23.0		N	mild	N	N	Y	56/22	5/2	6.8	<10	4.5	25	X		X		X	X		X	AF	VT

CABG = coronary artery bypass graft; MI/CAD = myocardial infarction/coronary artery disease; Y = yes, N = no;
PR = pulmonic valve regurgitation, MR = mitral valve regurgitation, AR = aortic valve regurgitation, TR = tricuspic valve regurgitation; DM = diabetes mellitus;
PAH = pulmonary artery hypertension, PAP = pulmonary artery pressure; ND = not determined; LVEDD = left ventricular end diastolic dimension, EF = ejection fraction;
ASA = acetylsalicylic acid, Amio = amiodarone, Mexil = mexiletine, ACE-I = angiotensin converting enzyme inhibitor;
AF = atrial fribrilation, SVT = supraventricular tachycardia, VF = ventricular fibrillation, VT = ventricular tachycardia; NSVT = nonsustained VT

Supplementary Table 4

MetaCore	IPA	Compiled	Overlapping
ABL1	ABL1	ADNP2	ATP2A2
ADNP2	ACOT11	ABL1	ABL1
ALDH3B1	AICAR	ACOT11	EIF2S1
ATP13A2	ALOX15	AICAR	ERO1A
ATP2A2	AMFR	ALDH3B1	NFE2L2
BMP4	AMPK	ALOX15	NOX4
BRF2	ANKS4B	AMFR	P4HB
CAT	APOBEC1	AMPK	PPARGC1A
CCS	AR	ANKS4B	SELENOS
CHCHD4	ATF3	APOBEC1	SOD1
COQ7	ATF4	AR	XBP1
CPEB2	ATF6	ATF3	
CST3	ATG10	ATF4	
CYBA	ATP2A1	ATF6	
CYBB	ATP2A2	ATG10	
CYCS	ATXN3	ATP13A2	
DHRS2	BAD	ATP2A1	
EIF2S1	BAK1	ATP2A2	
ENDOG	BAX	ATXN3	
ERO1A	BBC3	BAD	
ETV5	BBS12	BAK1	
FANCC	BCL2	BAX	
FANCD2	BCL2L11	BBC3	
FOXO1	BHLHA15	BBS12	
FOXO3	BID	BCL2	
G6PD	BIK	BCL2L11	
GGT1	BNIP3	BHLHA15	
GJB2	Calr4	BID	
GPX1	CALR	BIK	
GPX2	Casp12	BMP4	
GPX3	CCDC47	BNIP3	
GPX5	CCND1	BRF2	
GPX7	CD3	CALR	
GPX8	CD40LG	Calr4	
GSR	CDC37	Casp12	
GSTP1	CDK5RAP3	CAT	
HDAC1	CEBPB	CCDC47	
HDAC6	CFTR	CCND1	
HSPA1A	COL3A1	CCS	
HSPA1B	COL4A1	CD3	
HTRA2	COL4A3	CD40LG	
KAT2B	COL4A4	CDC37	
LONP1	COL4A3BP	CDK5RAP3	
LRRK2	COMP	CEBPB	
MGAT3	COPA	CFTR	
MGMT	CREB3	CHCHD4	
MSRB2	CREB3L1	COL3A1	
MSRB3	CREB3L2	COL4A1	
MT3	CREB3L3	COL4A3	
NCF1	CREBRF	COL4A3BP	
NCF2	CTH	COL4A4	
NCF4	CTSB	COMP	
NEFH	CTSD	COPA	
NFE2L1	CTSS	COQ7	
NFE2L2	CXCL8	CPEB2	
NGFR	Cyp2j9	CREB3	
NME1-NME2	DDIT3	CREB3L1	
NME2	DDRGK1	CREB3L2	
NOX4	DERL1	CREB3L3	
NOX5	DERL2	CREBRF	
NR4A2	DERL3	CST3	
NUDT2	DNAJA1	CTH	
P4HB	DNAJB1	CTSB	
PARK7	DNAJB2	CTSD	
PARP1	DNAJB4	CTSS	
PENK	DNAJB9	CXCL8	
PINK1	DNAJC4	CYBA	
PNPT1	DNAJC10	CYBB	
PPARGC1A	DST	CYCS	
PRDX1	EFEMP1	Cyp2j9	
PRDX2	EFEMP2	DDIT3	
PRDX3	EIF2A	DDRGK1	
PRDX5	EIF2AK2	DERL1	
PRDX6	EIF2AK3	DERL2	
PRKAA1	EIF2AK4	DERL3	
PRKAA2	EIF2B5	DHRS2	
PRKCD	EIF2S1	DNAJA1	
PRKD1	ELANE	DNAJB1	
PRKRA	EN460	DNAJB2	
PRR5L	EPHX2	DNAJB4	
PYCR1	ERN1	DNAJB9	
PYCR2	ERN2	DNAJC10	
PYROXD1	ERO1A	DNAJC4	
RAD52	ERP44	DST	
RBM11	FAM129A	EFEMP1	
RWDD1	FICD	EFEMP2	
SELENON	FLOT1	EIF2A	
SELENOS	GAA	EIF2AK2	
SESN2	GBA2	EIF2AK3	
SETX	GNRH	EIF2AK4	
SIRT2	GORASP2	EIF2B5	
SLC11A2	GPR37	EIF2S1	
SLC25A24	GSK3B	ELANE	
SLC7A11	HDL	EN460	
SNCA	HERPUD1	ENDOG	
SOD1	HFE	EPHX2	
SOD2	HMOX1	ERN1	
SOD3	Hsp90	ERN2	
SRXN1	HSP90AA1	ERO1A	
STAU1	HSP90AB1	ERP44	
STAU2	HSP90B1	ETV5	
TMEM161A	HSPA5	FAM129A	
TXN	HSPA1A/HSPA1B	FANCC	
TXN2	HSPA4L	FANCD2	
TXNDC2	HSPB3	FICD	
TXNDC8	HSPB7	FLOT1	

TXNL1	HSPD1	FOXO1	
TXNRD1	HSPE1	FOXO3	
TXNRD2	HSPH1	G6PD	
VKORC1L1	HTT	GAA	
VRK2	HYOU1	GBA2	
XBP1	IFNG	GGT1	
ZC3H12A	IGF1	GJB2	
ZFAND1	IGF2	GNRH	
	IL24	GORASP2	
	INS	GPR37	
	KDELR1	GPX1	
	LDL	GPX2	
	LRAT	GPX3	
	MAP3K5	GPX5	
	MAPK8	GPX7	
	MAPT	GPX8	
	MBTPS1	GSK3B	
	MBTPS2	GSR	
	MC4R	GSTP1	
	MFN2	HDAC1	
	MGAT2	HDAC6	
	MIA3	HDL	
	MMP9	HERPUD1	
	MPZ	HFE	
	MTOR	HMOX1	
	MTTP	Hsp90	
	MYH11	HSP90AA1	
	NFE2L2	HSP90AB1	
	NFkB (complex)	HSP90B1	
	NHLRC1	HSPA1A	
	NNAT	HSPA1A/HSPA1B	
	NOX4	HSPA1B	
	NRBF2	HSPA4L	
	OPN1SW	HSPA5	
	ORMDL3	HSPB3	
	OS9	HSPB7	
	P4HB	HSPD1	
	PARP16	HSPE1	
	PI3K (family)	HSPH1	
	PIK3C3	HTRA2	
	PIK3IP1	HTT	
	PIK3R1	HYOU1	
	PIK3R2	IFNG	
	PLA2G6	IGF1	
	PML	IGF2	
	PMP22	IL24	
	PNLIPRP2	INS	
	PPARGC1A	KAT2B	
	PPP1R15A	KDELR1	
	PPP1R15B	LDL	
	PPP2CB	LONP1	
	PRKAA	LRAT	
	PRKN	LRRK2	
	PSEN2	MAP3K5	
	PTPN1	MAPK8	
	PU-H71	MAPT	
	PXDNL	MBTPS1	
	QM295	MBTPS2	
	RAB6A	MC4R	
	RARA	MFN2	
	RASGRF1	MGAT2	
	RASGRF2	MGAT3	
	RIPK2	MGMT	
	RNF183	MIA3	
	RTN1	MMP9	
	SCAMP5	MPZ	
	SDF2L1	MSRB2	
	SEC16A	MSRB3	
	SEL1L	MT3	
	SELENOS	MTOR	
	SERP1	MTTP	
	SERPINB3	MYH11	
	SERPINH1	NCF1	
	SFTPC	NCF2	
	SIL1	NCF4	
	SIRT1	NEFH	
	SLC38A2	NFE2L1	
	SOD1	NFE2L2	
	SQSTM1	NFkB (complex)	
	SREBF1	NGFR	
	SRPX	NHLRC1	
	STC2	NME1-NME2	
	STIM1	NME2	
	STT3B	NNAT	
	STUB1	NOX4	
	SYVN1	NOX5	
	TBL2	NR4A2	
	TCR	NRBF2	
	THBS1	NUDT2	
	THBS4	OPN1SW	
	Tlr	ORMDL3	
	TMBIM6	OS9	
	TMCO1	P4HB	
	TMEM33	PARK7	
	TMEM259	PARP1	
	TMTC3	PARP16	
	TMX1	PENK	
	TNF	PI3K (family)	
	TNFRSF10B	PIK3C3	
	TOR1B	PIK3IP1	
	TP53	PIK3R1	
	TRIB3	PIK3R2	
	TRPC1	PINK1	
	TSC2	PLA2G6	
	UBA5	PML	
	UBE4B	PMP22	
	UBQLN1	PNLIPRP2	
	UCHL1	PNPT1	
	UFC1	PPARGC1A	
	UFL1	PPP1R15A	
	UFM1	PPP1R15B	
	VAPB	PPP2CB	

	VCP	PRDX1	
	WFS1	PRDX2	
	XBP1	PRDX3	
	YOD1	PRDX5	
	ZFYVE27	PRDX6	
		PRKAA	
		PRKAA1	
		PRKAA2	
		PRKCD	
		PRKD1	
		PRKN	
		PRKRA	
		PRR5L	
		PSEN2	
		PTPN1	
		PU-H71	
		PXDNL	
		PYCR1	
		PYCR2	
		PYROXD1	
		QM295	
		RAB6A	
		RAD52	
		RARA	
		RASGRF1	
		RASGRF2	
		RBM11	
		RIPK2	
		RNF183	
		RTN1	
		RWDD1	
		SCAMP5	
		SDF2L1	
		SEC16A	
		SEL1L	
		SELENON	
		SELENOS	
		SERP1	
		SERPINB3	
		SERPINH1	
		SESN2	
		SETX	
		SFTPC	
		SIL1	
		SIRT1	
		SIRT2	
		SLC11A2	
		SLC25A24	
		SLC38A2	
		SLC7A11	
		SNCA	
		SOD1	
		SOD2	
		SOD3	
		SQSTM1	
		SREBF1	
		SRPX	
		SRXN1	
		STAU1	
		STAU2	
		STC2	
		STIM1	
		STT3B	
		STUB1	
		SYVN1	
		TBL2	
		TCR	
		THBS1	
		THBS4	
		Tlr	
		TMBIM6	
		TMCO1	
		TMEM161A	
		TMEM259	
		TMEM33	
		TMTC3	
		TMX1	
		TNF	
		TNFRSF10B	
		TOR1B	
		TP53	
		TRIB3	
		TRPC1	
		TSC2	
		TXN	
		TXN2	
		TXNDC2	
		TXNDC8	
		TXNL1	
		TXNRD1	
		TXNRD2	
		UBA5	
		UBE4B	
		UBQLN1	
		UCHL1	
		UFC1	
		UFL1	
		UFM1	
		VAPB	
		VCP	
		VKORC1L1	
		VRK2	
		WFS1	
		XBP1	
		YOD1	
		ZC3H12A	
		ZFAND1	
		ZFYVE27	

Supplementary Table 5

MetaCore	IPA	Compiled	Overlapping
ABL1	ABCB10	ADNP2	ABL1
ADNP2	ABCC1	ABCB10	ATP2A2
ALDH3B1	ABCG2	ABCC1	CAT
ATP13A2	ABL1	ABCG2	COQ7
ATP2A2	ACE2	ABL1	CST3
BMP4	ACE	ACE	CYBB
BRF2	ACOT11	ACE2	FOXO3
CAT	ACS84	ACOT11	G6PD
CCS	ADIPOQ	ACS84	GPX1
CHCHD4	ADM	ADIPOQ	GPX7
COQ7	AG490	ADM	LRRK2
CPEB2	AGT	AG490	NFE2L1
CST3	AHR	AGT	NFE2L2
CYBA	Ahsp	AHR	NGFR
CYBB	AIFM1	Ahsp	NOX4
CYCS	ALDH1A1	AIFM1	NR4A2
DHRS2	ALDH3A2	ALDH1A1	PARK7
EIF2S1	ALOX12	ALDH3A2	PINK1
ENDOG	ALS2	ALDH3B1	PPARGC1A
ERO1A	Ang2	ALOX12	PRDX1
ETV5	ANGPT2	ALS2	PRDX2
FANCC	ANGPTL7	Ang2	PRDX3
FANCD2	APOE	ANGPT2	PRDX5
FOXO1	APP	ANGPTL7	PRDX6
FOXO3	ATF4	APOE	PRKCD
G6PD	ATOX1	APP	SNCA
GGT1	ATP2A2	ATF4	SOD1
GJB2	ATRN	ATOX1	SOD2
GPX1	BAK1	ATP13A2	SRXN1
GPX2	BAX	ATP2A2	TXN
GPX3	BCKDK	ATRN	TXNDC2
GPX5	BCL2	BAK1	TXNRD1
GPX7	BRCA1	BAX	
GPX8	C19orf12	BCKDK	
GSR	CA3	BCL2	
GSTP1	CA5A	BMP4	
HDAC1	CA5B	BRCA1	
HDAC6	CAMK2G	BRF2	
HSPA1A	CAT	C19orf12	
HSPA1B	CBS/CBSL	CA3	
HTRA2	CDK5RAP1	CA5A	
KAT2B	CHRNA4	CA5B	
LONP1	CLEC12A	CAMK2G	
LRRK2	COMT	CAT	
MGAT3	COPA	CBS/CBSL	
MGMT	COQ7	CCS	
MSRB2	COQ9	CDK5RAP1	
MSRB3	CST3	CHCHD4	
MT3	CYB5R3	CHRNA4	
NCF1	CYB5R4	CLEC12A	
NCF2	CYBB	COMT	
NCF4	CYGB	COPA	
NEFH	CYP2E1	COQ7	
NFE2L1	DDIT3	COQ9	
NFE2L2	DGKK	CPEB2	
NGFR	DHODH	CST3	
NME1-NME2	DIABLO	CYB5R3	
NME2	DKC1	CYB5R4	
NOX4	DNAJB9	CYBA	
NOX5	DOCA/HS	CYBB	
NR4A2	EFEMP2	CYCS	
NUDT2	EIF2AK4	CYGB	
P4HB	EPAS1	CYP2E1	
PARK7	ERCC1	DDIT3	
PARP1	ERCC2	DGKK	
PENK	ERCC3	DHODH	
PINK1	ERCC6	DHRS2	
PNPT1	ERCC8	DIABLO	
PPARGC1A	ERK1/2	DKC1	
PRDX1	ETFDH	DNAJB9	
PRDX2	FABP1	DOCA/HS	
PRDX3	FBXL5	EFEMP2	
PRDX5	FGF8	EIF2AK4	
PRDX6	FOXO3	EIF2S1	
PRKAA1	FTH1	ENDOG	
PRKAA2	G6PD	EPAS1	
PRKCD	GAB1	ERCC1	
PRKD1	GADD45	ERCC2	
PRKRA	GCLC	ERCC3	
PRR5L	GCLM	ERCC6	
PYCR1	GDAP1	ERCC8	
PYCR2	GGT5	ERK1/2	
PYROXD1	GMFB	ERO1A	
RAD52	GPD2	ETFDH	
RBM11	GPX1	ETV5	
RWDD1	GPX4	FABP1	
SELENON	GPX7	FANCC	
SELENOS	GRK2	FANCD2	
SESN2	GSS	FBXL5	
SETX	Gsta4	FGF8	
SIRT2	GSTZ1	FOXO1	
SLC11A2	HDL	FOXO3	
SLC25A24	HFE	FTH1	
SLC7A11	HINT2	G6PD	
SNCA	HMOX1	GAB1	
SOD1	HMOX2	GADD45	
SOD2	HNF1A	GCLC	
SOD3	HSD17B10	GCLM	
SRXN1	Hsp27	GDAP1	
STAU1	Hsp70	GGT1	
STAU2	HSPA9	GGT5	
TMEM161A	HSPB1	GJB2	
TXN	HSPB2	GMFB	
TXN2	HTT	GPD2	
TXNDC2	IDH1	GPX1	
TXNDC8	IGF2	GPX2	
TXNL1	IL33	GPX3	
TXNRD1	INSR	GPX4	
TXNRD2	IPCEF1	GPX5	
VKORC1L1	ISCU	GPX7	
VRK2	JAK2	GPX8	
XBP1	JAK	GRK2	
ZC3H12A	JUN	GSR	
ZFAND1	KCNA1	GSS	
	KLC1	Gsta4	
	LANCL1	GSTP1	
	LDLR	GSTZ1	

	LEP	HDAC1	
	LGALS3	HDAC6	
	LIAS	HDL	
	LRAT	HFE	
	LRRK2	HINT2	
	MAOB	HMOX1	
	MAP2K1	HMOX2	
	MAPK14	HNF1A	
	MAPT	HSD17B10	
	MEIS1	Hsp27	
	MET	Hsp70	
	MFN2	HSPA1A	
	MICB	HSPA1B	
	MMP2	HSPA9	
	MMP9	HSPB1	
	MPO	HSPB2	
	Mpo	HTRA2	
	MSRA	HTT	
	MSTN	IDH1	
	Mt1	IGF2	
	Mt2	IL33	
	Mt3	INSR	
	MT-CO1	IPCEF1	
	MT-ND3	ISCU	
	MTF1	JAK	
	MUC1	JAK2	
	MUTYH	JUN	
	MYC	KAT2B	
	MYH11	KCNA1	
	NAMPT	KLC1	
	NAPRT	LANCL1	
	NDUFA6	LDLR	
	NDUFA12	LEP	
	NDUFB4	LGALS3	
	NDUFS2	LIAS	
	NDUFS4	LONP1	
	NDUFS8	LRAT	
	NEIL1	LRRK2	
	NEIL2	MAOB	
	NFE2L1	MAP2K1	
	NFE2L2	MAPK14	
	NFKB1	MAPT	
	NGFR	MEIS1	
	NME8	MET	
	NOS2	MFN2	
	NOS3	MGAT3	
	NOX4	MGMT	
	NQO1	MICB	
	NR3C2	MMP2	
	NR4A2	MMP9	
	NR4A3	MPO	
	NRF1	MSRA	
	NRG (family)	MSRB2	
	NRROS	MSRB3	
	NUDT1	MSTN	
	OGDH	MT-CO1	
	OGG1	MT-ND3	
	OPN1SW	Mt1	
	ORAI1	Mt2	
	OXR1	MT3	
	OXSR1	MTF1	
	PARK7	MUC1	
	PCLAF	MUTYH	
	PDHA1	MYC	
	PDLIM1	MYH11	
	PDSS2	NAMPT	
	PEMT	NAPRT	
	PEX13	NCF1	
	PEX11B	NCF2	
	PI3K (complex)	NCF4	
	PIK3R1	NDUFA12	
	PINK1	NDUFA6	
	PLIN5	NDUFB4	
	PNKP	NDUFS2	
	PON1	NDUFS4	
	PON2	NDUFS8	
	PON3	NEFH	
	PPARG	NEIL1	
	PPARGC1A	NEIL2	
	PPARGC1B	NFE2L1	
	PPID	NFE2L2	
	PPIF	NFKB1	
	PPP1R15B	NGFR	
	PRDX1	NME1-NME2	
	PRDX2	NME2	
	PRDX3	NME8	
	PRDX4	NOS2	
	PRDX5	NOS3	
	PRDX6	NOX4	
	PRKCD	NOX5	
	PRKN	NQO1	
	PRNP	NR3C2	
	PSEN1	NR4A2	
	PSIP1	NR4A3	
	PSMB5	NRF1	
	PTEN	NRG (family)	
	PTGS1	NRROS	
	PTGS2	NUDT1	
	QDPR	NUDT2	
	RAC1	OGDH	
	RBP1	OGG1	
	RBPMS	OPN1SW	
	RCAN1	ORAI1	
	RCAN2	OXR1	
	REN	OXSR1	
	RGN	P4HB	
	RGS14	PARK7	
	RIPK2	PARP1	
	RPS3	PCLAF	
	RRM2B	PDHA1	
	RXRA	PDLIM1	
	S100A12	PDSS2	
	SB203580	PEMT	
	SCARA3	PENK	
	SELENOF	PEX11B	
	SELENOK	PEX13	
	SELENOP	PI3K (complex)	
	SERPIND1	PIK3R1	
	SGK2	PINK1	
	SGMS1	PLIN5	
	SHC1	PNKP	
	SIL1	PNPT1	
	SIRT1	PON1	
	SIRT3	PON2	

	SNCA	PON3	
	SNPH	PPARG	
	SOD1	PPARGC1A	
	SOD2	PPARGC1B	
	SQSTM1	PPID	
	SRXN1	PPIF	
	STC2	PPP1R15B	
	STEAP4	PRDX1	
	STIM1	PRDX2	
	STK25	PRDX3	
	TARDBP	PRDX4	
	TAT	PRDX5	
	TGFB1	PRDX6	
	TLR4	PRKAA1	
	TNF	PRKAA2	
	TOP1MT	PRKCD	
	TOR1A	PRKD1	
	TP53	PRKN	
	TP53INP1	PRKRA	
	TRPM2	PRNP	
	TXN	PRR5L	
	TXNDC2	PSEN1	
	TXNIP	PSIP1	
	TXNRD1	PSMB5	
	UBE4B	PTEN	
	UCN	PTGS1	
	UCP3	PTGS2	
	USP10	PYCR1	
	VASN	PYCR2	
	VAV1	PYROXD1	
	VHL	QDPR	
	VNN1	RAC1	
	WRN	RAD52	
	XDH	RBM11	
	XPA	RBP1	
	XPC	RBPMS	
	YBX1	RCAN1	
	ZFP36	RCAN2	
		REN	
		RGN	
		RGS14	
		RIPK2	
		RPS3	
		RRM2B	
		RWDD1	
		RXRA	
		S100A12	
		SB203580	
		SCARA3	
		SELENOF	
		SELENOK	
		SELENON	
		SELENOP	
		SELENOS	
		SERPIND1	
		SESN2	
		SETX	
		SGK2	
		SGMS1	
		SHC1	
		SIL1	
		SIRT1	
		SIRT2	
		SIRT3	
		SLC11A2	
		SLC25A24	
		SLC7A11	
		SNCA	
		SNPH	
		SOD1	
		SOD2	
		SOD3	
		SQSTM1	
		SRXN1	
		STAU1	
		STAU2	
		STC2	
		STEAP4	
		STIM1	
		STK25	
		TARDBP	
		TAT	
		TGFB1	
		TLR4	
		TMEM161A	
		TNF	
		TOP1MT	
		TOR1A	
		TP53	
		TP53INP1	
		TRPM2	
		TXN	
		TXN2	
		TXNDC2	
		TXNDC8	
		TXNIP	
		TXNL1	
		TXNRD1	
		TXNRD2	
		UBE4B	
		UCN	
		UCP3	
		USP10	
		VASN	
		VAV1	
		VHL	
		VKORC1L1	
		VNN1	
		VRK2	
		WRN	
		XBP1	
		XDH	
		XPA	
		XPC	
		YBX1	
		ZC3H12A	
		ZFAND1	
		ZFP36	

Supplementary Table 6

	Treatment											
	Saline						Kainic Acid (30 mg/kg body weight)					
**Sample**	1	2	3	4	5	6	7	8	9	10	11	12
**Weight (g)**	23.5	22	21	20	20	20	20.4	21	23	19	22	22
**Behavior Score (average)**	0	0	0	0	0	0	2.4615	3.5385	2.9231	3.3077	5.4615	5.4615

Supplementary Table 7

Donor ID	Tumor Name	Age (yrs)	Survival Days	Surgery	Molecular Subtype
12111	W10-1-1	44		primary	Proneural
12112	W11-1-1	57	1076	primary	Classical, Mesenchymal
10865	W1-1-2	66	105	primary	Classical
12165	W12-1-1	61	80	primary	Classical
12877	W13-1-1	59	250	primary	Mesenchymal
12996	W16-1-1	76	353	primary	Neural, Proneural
159992499	W18-1-1	36	903	primary	
159994896	W19-1-1	67	615	primary	Proneural
277805907	W20-2-1	43	363	recurrent	Mesenchymal
10926	W2-1-1	64	1096	primary	Classical, Neural
12995	W21-1-1	54	446	primary	Proneural
12997	W22-1-1	52		primary	Classical, Neural
268091685	W22-2-1	52		recurrent	Neural
13988	W26-1-1	57	1293	primary	Neural
277857061	W27-2-1	64	72	recurrent	Classical
14220	W28-1-1	68	300	primary	Mesenchymal, Neural
14221	W29-1-1	73	260	primary	Classical, Neural
14545	W30-1-1	59	759	primary	
12102	W3-1-1	65	982	primary	Classical, Mesenchymal
14734	W31-1-1	17	871	primary	Proneural
14737	W32-1-1	56		primary	Proneural
14738	W33-1-1	60		primary	Classical
14762	W34-1-1	73	351	primary	Classical, Mesenchymal
292023102	W35-1-1	36		primary	
14763	W36-1-1	61	544	primary	Mesenchymal
15343	W38-1-1	64	311	primary	Proneural
15350	W39-1-1	67	880	primary	Classical
15400	W40-1-1	64	184	primary	Neural
12106	W4-1-1	50	540	primary	Mesenchymal, Neural
113597455	W42-1-1	59	197	primary	Neural, Proneural
113597961	W43-1-1	61	300	primary	Mesenchymal, Neural
159446123	W45-1-1	63	542	primary	
161243620	W48-1-1	51	456	primary	Neural
180682564	W50-1-1	26		primary	
12109	W5-1-1	64		primary	Classical, Neural
268091642	W53-1-1	54		primary	Classical
286825896	W54-1-1	57	62	primary	Proneural
292023109	W55-1-1	52		primary	Classical
12107	W6-1-1	72	633	primary	Mesenchymal
12105	W7-1-1	60	437	primary	Mesenchymal
12108	W8-1-1	49	442	primary	Classical, Mesenchymal
12110	W9-1-1	50	145	primary	Proneural
